# Functional Characterization of a Regiospecific Sugar-*O*-Methyltransferase from *Nocardia*

**DOI:** 10.1128/aem.00754-22

**Published:** 2022-06-15

**Authors:** Purna Bahadur Poudel, Ramesh Prasad Pandey, Dipesh Dhakal, Tae-Su Kim, Trang Thi Huyen Nguyen, Hye Jin Jung, Hee Jeong Shin, Binod Timalsina, Jae Kyung Sohng

**Affiliations:** a Institute of Biomolecule Reconstruction (iBR), Department of Life Science and Biochemical Engineering, Sun Moon University, Asan-si, Chungnam, Republic of Korea; b Department of Biotechnology and Pharmaceutical Engineering, Sun Moon University, Asan-si, Chungnam, Republic of Korea; c Department of Anatomy, Dongguk University College of Medicine, Gyeongju, Republic of Korea; University of Illinois at Urbana-Champaign

**Keywords:** *O*-methyltransferase, regiospecific, *Nocardia*, anthraquinone

## Abstract

Methyltransferases transfer a methyl group to a diverse group of natural products, thus providing structural diversity, stability, and altered pharmacological properties to the molecules. A limited number of regiospecific sugar-*O*-methyltransferases are functionally characterized. Thus, discovery of such an enzyme could solve the difficulties of biological production of methoxy derivatives of glycosylated molecules. In the current study, a regiospecific sugar-*O*-methyltransferase, ThnM1, belonging to the biosynthetic gene cluster (BGC) of 1-(α-L-(2-*O*-methyl)-6-deoxymannopyranosyloxy)-3,6,8-trimethoxynaphthalene produced by *Nocardia* sp. strain CS682, was analyzed and functionally characterized. ThnM1 demonstrated promiscuity to diverse chemical structures such as rhamnose-containing anthraquinones and flavonoids with regiospecific methylation at the 2′-hydroxyl group of the sugar moiety. Compared with other compounds, anthraquinone rhamnosides were found to be the preferred substrates for methylation. Thus, the enzyme was further employed for whole-cell biotransformation using engineered Escherichia coli to produce a methoxy-rhamnosyl derivative of quinizarin, an anthraquinone derivative. The structure of the newly generated derivative from Escherichia coli fermentation was elucidated by liquid chromatography-mass spectrometry and nuclear magnetic resonance spectroscopic analyses and identified as quinizarin-4-*O*-α-l-2-*O*-methylrhamnoside (QRM). Further, the biological impact of methylation was studied by comparing the cytotoxicity of QRM with that of quinizarin against the U87MG, SNU-1, and A375SM cancer cell lines.

**IMPORTANCE** ThnM1 is a putative sugar-*O*-methyltransferase produced by the *Nocardia* sp. strain CS682 and is encoded by a gene belonging to the biosynthetic gene cluster (BGC) of 1-(α-l-(2-*O*-methyl)-6-deoxymannopyranosyloxy)-3,6,8-trimethoxynaphthalene. We demonstrated that ThnM1 is a promiscuous enzyme with regiospecific activity at the 2′-OH of rhamnose. As regiospecific methylation of sugars by chemical synthesis is a challenging step, ThnM1 may fill the gap in the potential diversification of natural products by methylating the rhamnose moiety attached to them.

## INTRODUCTION

The chemical and structural diversities found in natural products (NPs) have been a great resource for designing molecules as pharmacophores for drug discovery. In addition to chemical synthesis, molecules can also be diversified by enzymatic modification reactions such as hydroxylation, glycosylation, methylation, sulfation, prenylation, and halogenation (1–7). Such modifications of NPs provide a vast array of structural diversity to the molecule and can dramatically alter its pharmacological properties and modes of action (8–12). Among the numerous modifications, methylation is an NP postbiosynthesis reaction, which is mediated by a methyltransferase (MT) enzyme. MT catalyzes the transfer of the methyl group from donors such as *S*-adenosyl-l-methionine (SAM), methylcobalamin, and methyltetrahydrofolate to acceptor groups containing S, N, O, or C atoms (13–17). SAM-dependent MTs are a large superfamily whose members have the same folding topology for binding SAM in an identical manner. These enzymes are thought to have diverged from the same MT ancestor as DNA MTs; however, many branches of this superfamily have highly divergent sequences (13, 14, 18, 19). The biosynthesis pathway of 1-(α-l-(2-*O*-methyl)-6-deoxymannopyranosyloxy)-3,6,8-trimethoxynaphthalene), a UV-protection compound produced by *Nocardia* sp. strain CS682 (20), contains three putative *O*-methyltransferases, ThnM1, ThnM2, and ThnM3. The α-sugars in NPs, such as doxorubicin (an anticancer compound) (8), erythromycin (an antibacterial compound) (21), spinosyn (an insecticidal compound) (22), and avermectin (an antihelminth compound) (23), are attributed to the biological activities of the parent molecules. In particular, 2-*O*-methyl-l-rhamnose is a constituent of several bioactive molecules, such as scopamycin A (a metabolite produced by a Streptomyces aureofaciens strain [24]), aranciamycin (an antibiotic produced by a Streptomyces echinatus [25]), and 1-(α-l-(2-*O*-methyl)-6-deoxymannopyranosyloxy)-3,6,8-trimethoxynaphthalene (a UV-protective compound produced by *Nocardia* sp. CS682 (20). The regiospecific chemical synthesis of *O*-methylated sugars is challenging and unsustainable because it requires (i) harsh catalytic conditions, (ii) the release of toxic by-products, (iii) a long reaction time, (iv) protection/deprotection of unwanted groups, and (v) expensive reagents. Nevertheless, biocatalytic methylation using enzymes and engineered microorganisms are capable of *O*-methylation of compounds for producing novel bioactive compounds in a much milder and single-step reaction using resources from microbial cells (26, 27).

In our early attempts to characterize regiospecific MTs for sugars, enzymes were found to be equally reactive toward the aglycon, limiting their application in the regiospecific production of methoxy derivatives of glycosylated molecules (1, 28). In this study, we identified a new sugar *O*-methyltransferase, ThnM1, which transfers a methyl group to rhamnosylated polyphenolic compounds. As a proof of concept, ThnM1 was applied for the biosynthesis of the methoxy derivative of quinizarin-4-*O*-α-l-rhamnoside (QR), which was found to be the most preferred substrate among the tested NPs in *in vitro* reactions. We also generated an engineered Escherichia coli strain to enhance the pool of SAM for the optimal production of quinizarin-4-*O*-α-l-methylrhamnoside, starting from exogenously supplemented quinizarin.

## RESULTS AND DISCUSSION

### Sequence and phylogenetic analysis of ThnM1.

**FIG 1:**
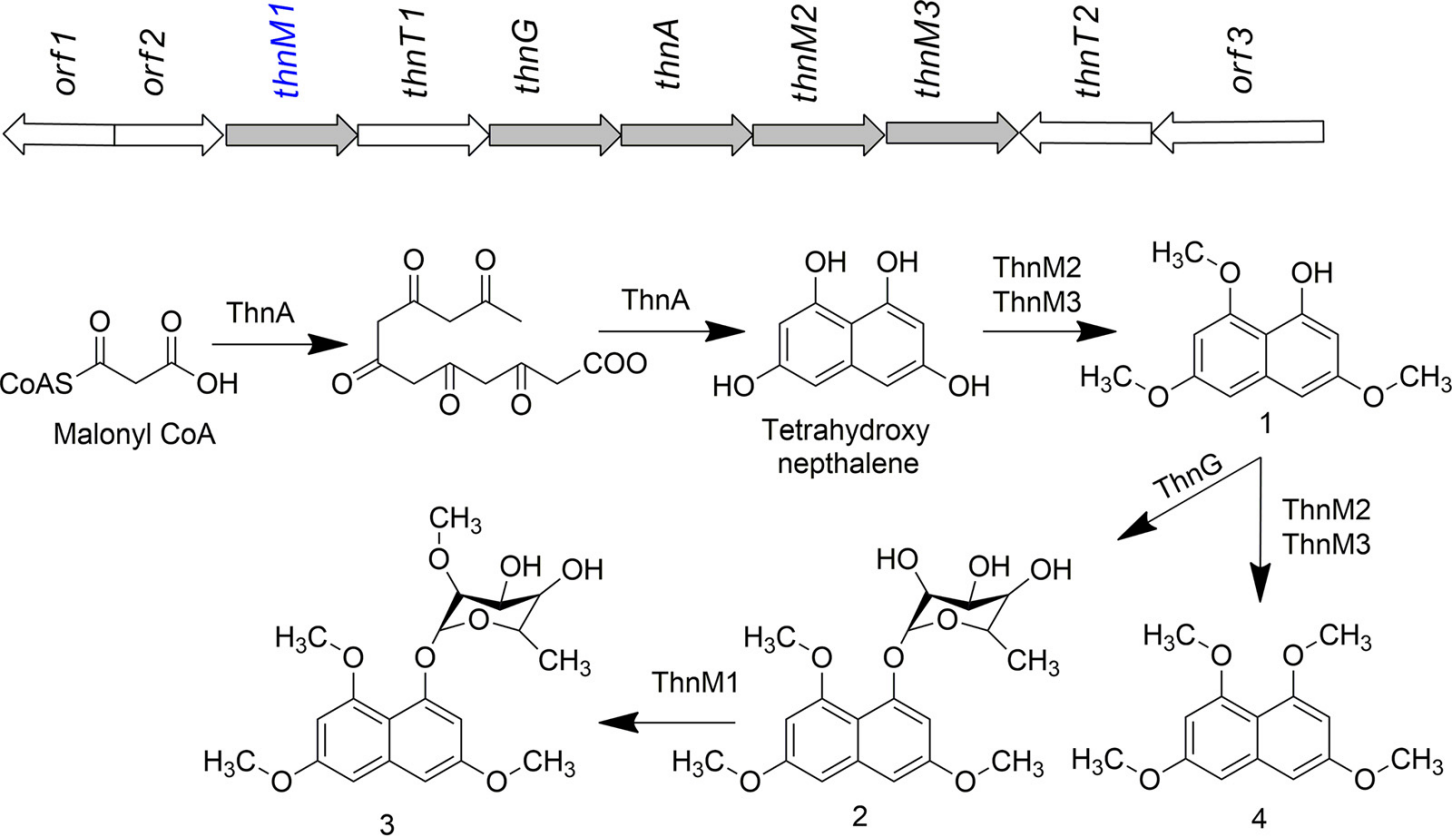
The putative biosynthetic gene cluster and proposed biosynthetic pathway of compound 3. (Compound 1) 3,6,8-trimethoxy naphthalen-1-ol; (2) 1-(α-l-6-deoxy-mannopyranosyloxy)-3,6,8-trimethoxy naphthalene; (3) 1-(α-l-(2-*O*-methyl)-6-deoxymanno-pyranosyloxy)-3,6,8-trimethoxynaphthalene; (4) 1,3,6,8-tetramethoxynaphthalene.

Genome analysis showed that the biosynthetic gene cluster (BGC) of 1-(α-l-(2-*O*-methyl)-6-deoxymannopyranosyloxy)-3,6,8-trimethoxynaphthalene includes three *O*-methyltransferases: ThnM1, ThnM2, and ThnM3 (Fig. 1). These MTs are putatively annotated for methylation of either the hydroxyl group of 1,3,6,8-tetrahydroxnaphtalene (THN) or the sugar moiety attached to THN to produce intermediates or the final metabolite (20).

Phylogenetic tree analysis of ThnM1 and other MTs from diverse actinomycetes (see Fig. S1 in the supplemental material) showed that ThnM1 falls in a clade distinct from the previously characterized sugar-*O*-methyltransferases. However, it shared high sequence similarity (51% to 55% amino acid sequence) with them, such as MycE, ElmM1, ElmM2, OleY, GerMIII, Biki, TylE, and BusK (Fig. S2). Amino acid sequence analysis also demonstrated that ThnM1 has a highly conserved active site domain comprising Tyr208, Asp275, His278, and Asp304 (Fig. S2).

### Cloning, expression, and purification of ThnM1.

The gene *thnM1* was cloned into the pET-32a(+) expression vector under the T7 promoter and transformed into the E. coli BL21(DE3) expression host. The SDS-PAGE analysis of the clear lysate, unclear lysate, and insoluble fraction of the protein along with the purified fraction of the enzyme showed bands corresponding to the theoretical molecular weight, 42.7 kDa, and a single band at ~63 kDa [with a His tag from the pET-32a(+) vector] (Fig. S3). Most fractions of the protein were present in the clear lysate, whereas a fraction of the protein was also present in the inclusion bodies. The clear lysate was used for the purification of His-tagged ThnM1 protein using TALON cobalt resin. The purified fractions of the protein were pooled and used for further quantification and studies, as described in Materials and Methods.

### Substrate promiscuity of ThnM1.

1-(α-l-6-deoxy-mannopyranosyloxy)-3,6,8-trimethoxy naphthalene is one of the metabolites produced by *Nocardia* sp. CS682, and BGC analysis showed that ThnM1 lies within the pathway (Fig. 1). Thus, the aforementioned molecule is a natural substrate for ThnM1. However, owing to the commercial unavailability of this molecule, we used several other compounds that are available in the laboratory as pseudosubstrates for testing; these included diverse classes of synthetic molecules that resemble the structure of the core moiety of the metabolite 1-(α-l-6-deoxy-mannopyranosyloxy)-3,6,8-trimethoxy naphthalene. Moreover, other plants and microbial metabolites, along with their sugar-conjugated derivatives, were assessed for *in vitro* enzymatic reactions using the same enzyme.

**FIG 2:**
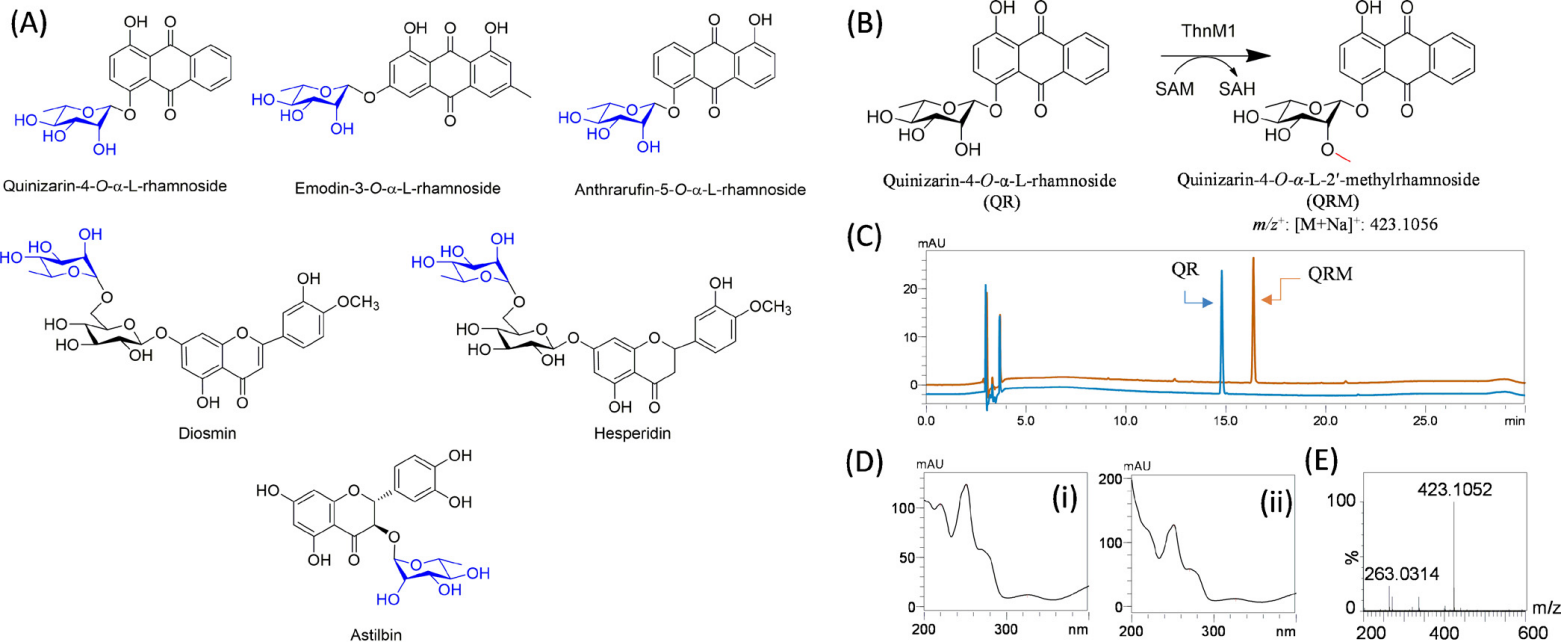
(A) Structures of different substrates that are accepted by ThnM1in *in vitro* reactions. (B) Reaction scheme of methylation of quinizarin-4-*O*-α-l-rhamnoside (QR) by ThnM1 in the presence of SAM at 40°C for 3 h (50 mM Tris HCl buffer [pH 7.5], 2 mM SAM, 2 mM substrate, 2 mM MgCl_2_, and 50 μg of ThnM1 protein). (C) HPLC-PDA chromatogram of quinizarin-4-*O*-α-l-rhamnoside (QR) reaction with ThnM1 to form the product quinizarin-4-*O*-α-l-2′-*O*-methylrhamnoside (QRM). (D, i) UV/visible (Vis) of QR; (ii) UV/Vis of QRM. (E) HR-QTOF ESI/MS spectrum for methylated product of QR.

The results showed that ThnM1 could not methylate any of the synthetic naphthalene derivatives, such as 1,2-dihydronaphthalene, 1,5-dihydronaphthalene, 1,6-dihydronaphthalene, 1,7-dihydronaphthalene, 1,8-dihydronaphthalene, 2,3-dihydronaphthalene, and 2,7-dihydronaphthalene, which are structurally partially similar to the aglycone part of the 1-(α-l-6-deoxy-mannopyranosyloxy)-3,6,8-trimethoxynaphthalene (Fig. S4). Furthermore, the enzyme was also found to be incapable of catalyzing the methylation reaction of other metabolites, such as anthraquinones (alizarin, emodin, anthrarufin, chrysazin/dantron, and quinizarin) and larger polyketides, such as nargenicin (Fig. S4), which is also a major metabolite produced by the same strain of *Nocardia*. Because none of the aglycone molecules were methylated by ThnM1, we speculated that ThnM1 is most likely a sugar-*O*-methyltransferase that transfers a methyl group to the sugar moieties. We hence screened the enzyme for other glycosylated molecules available to us. Interestingly, when ThnM1 reacted with some rhamnose moieties containing derivatives of anthraquinones and flavonoids, it successfully catalyzed the methylation reaction with QR, emodin-3-*O*-α-l-rhamnoside (ER), anthrarufin-5-*O*-α-l-rhamnoside (AR), and flavonoids (astilbin, hesperidin, and diosmin) (Fig. 2A). The reaction mixtures were analyzed by high-performance liquid chromatography (HPLC) and LC coupled with high-resolution quadrupole time-of-flight electrospray ionization-mass spectrometry (HR-QTOF ESI/MS). The HPLC-photodiode array (PDA) revealed that the ThnM1-catalyzed QR reaction resulted in a single prominent product peak at retention time (*t*_R_) 16.4 min, after the substrate peak at *t*_R_ 14.8 min, when measured at 420 nm (the maximum UV absorbance [λ_max_] for quinizarin) (Fig. 2B to D). We further analyzed the same samples using HR-QTOF ESI/MS. The calculated mass of *O*-methylated QR matched the measured mass ([M+Na]^+^ *m/z*^+^ 423.1056) in the positive mode in the sodium adduct (Fig. 2E). The methylated products of other rhamnosylated anthraquinones and flavonoids were confirmed by following the same protocol (Fig. S5 to S9).

To determine the promiscuity of ThnM1 with glucosylated molecules, such as naringenin 7-*O*-β-d-glucoside and naringenin 4-*O*-β-d-glucoside, identical reactions were carried out. However, no products were detected corresponding to these substrates (Fig. S4). Nevertheless, hesperidin and diosmin, which contain a disaccharide composed of a glucose that is directly conjugated to the backbone of flavonoids and a rhamnose that is conjugated over glucose by a glycosidic bond, are methylated by ThnM1 to produce its methylated derivatives (Fig. S8, and S9).

Among different substrates tested, QR showed 95% conversion, followed by ER and AR, with 15% conversion. However, the conversion percentages of diosmin, astilbin, and hesperidin were 6%, 5%, and 5%, respectively (Fig. S10). Since QR exhibited the highest conversion with ThnM1, we chose it as a substrate to characterize the enzyme’s catalytic properties.

### Effects of incubation temperature, pH, and cofactors on enzyme activity.

The effects of incubation temperature, pH, and cofactors on the activity of ThnM1 involving the conversion of QR were determined as described in Materials and Methods. ThnM1 exhibited the highest methylation activity at 40°C (Fig. S11A), with optimum activity at pH 7.5 (Fig. S11C). Interestingly, among the different metal cations tested, each at a concentration of 2 mM, Cu^2+^, Ca^2+^, Co^2+^, Fe^2+^, Zn^2+^, and Ni^2+^ rendered ThnM1 virtually inactive. However, the highest rate of QR conversion was observed with Mg^2+^, whereas that of Mn^2+^ was approximately half of that (Fig. S11B). The product observed in the control might be mediated by divalent cations copurified along with the protein because the medium used for cell growth was rich in divalent salts. This hypothesis was further supported by an experiment in which EDTA was used to determine the ThnM1 activity. EDTA quenched divalent metal ions, thus showing a significant loss of the ThnM1 activity (<1%) when EDTA (2 mM) was used in the reaction. This corroborates the divalent dependency of ThnM1 for its enzymatic function. Such metal dependency is a common feature of class II plant *O*-methyltransferases (1).

**FIG 3:**
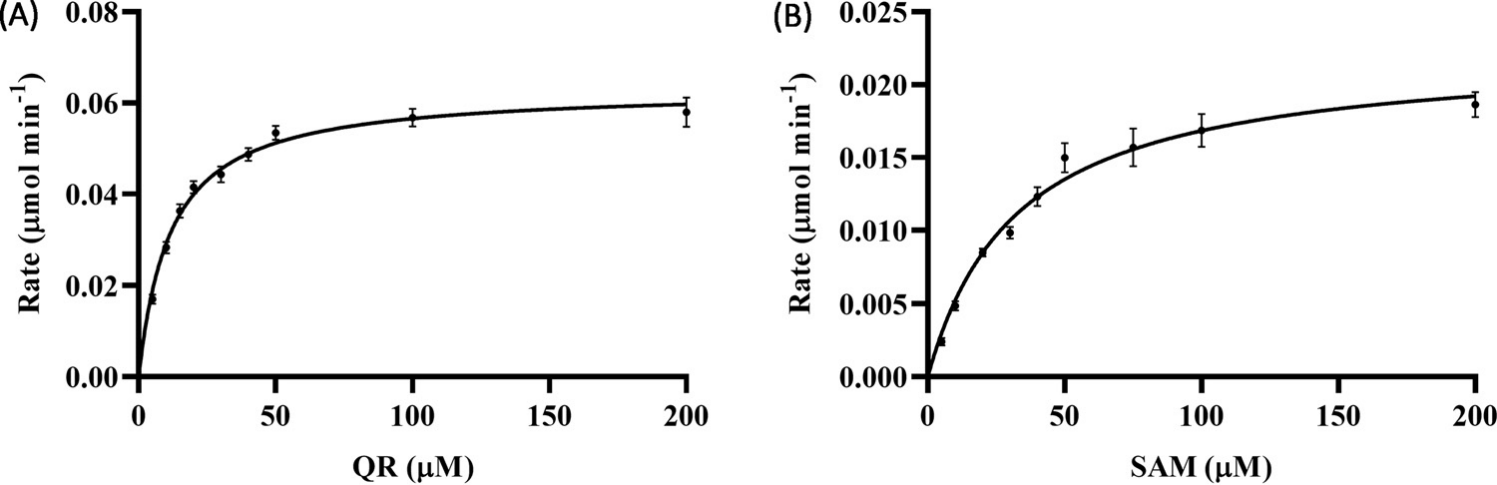
Enzyme-kinetics characteristics of ThnM1. (A) Kinetic analysis using various concentrations of QR (5 to 200 μM), ThnM1 (2 μg), SAM (2 mM), MgCl_2_ (2 mM), and Tris-HCl buffer (50 mM, pH 7.5). (B) Kinetic analysis using various concentrations of SAM (5 to 200 μM), ThnM1 (2 μg), QR (100 μM), MgCl_2_ (2 mM), and Tris-HCl buffer (50 mM, pH 7.5).

Finally, the steady-state kinetic parameters of ThnM1 were determined. Initial velocity was measured with 2 μg ThnM1 in a time period of 10 min after knowing the effect of enzyme concentration and a time course study (Fig. S11D and E). With saturating SAM (2 mM), an apparent *K_m_* value for QR was estimated to be 11.70 ± 1.97 μM (Fig. 3A). Near the saturation of QR (100 μM), the *K_m_* value for SAM was estimated to be 32.7 ± 7.06 μM (Fig. 3B). The apparent *K_m_* and *V*_max_ values are shown in Table S3.

### Reaction mechanism.

Biochemical reaction analysis showed that ThnM1 is responsible for the methylation of rhamnose moiety attached to diverse molecules, including flavonoids and anthraquinones. Based on MycE, the closest MT for which the crystal structure is known, it contains two water molecules and two aspartates (Asp275 and Asp304) that interact with Mg^++^ ions by octahedral coordination (29) (Fig. S12). In 6-deoxyallose, the 2′- and 3′-OH groups of mycinamicin VI interacted with the Mg^++^ ion, which bound to the active site domain of the two aspartates. Thus, the conformation of sugar 2′- and 3′-OH groups is very important for binding with Mg^++^ ions and the subsequent enzyme catalysis. These two aspartates are well conserved in MycE and ThnM1 (Fig. S2). Therefore, ThnM1 and MycE belong to the same family of metal-dependent NP sugar *O*-methyltransferases. In the case of allose and rhamnose, the 2′- and 3′-OH groups had the same conformation, making it easy to bind to Mg^++^ in the active site domain. The conformation of the 2′- and 3′-OH groups of sugars is essential for the methylation activity of ThnM1. Hence, ThnM1 can only methylate the 2′-OH group of rhamnose in anthraquinones and flavonoids. The second crucial factor that could play a role in affecting the activity of ThnM1 is the structure of the aglycone molecule. Among all substrates tested, QR was the most preferred substrate, with a conversion rate of 95%, followed by other rhamnosylated anthraquinones such as ER and AR (~15%) and glycosylated flavonoids such as astilbin, hesperidin, and diosmin (~5% to 6%) (Table 1 and Fig. S10). In anthraquinone, the ABC ring is connected in parallel; however, in flavonoids, the B ring is bonded at the 2′ position of the C ring by the C2-1 carbon-carbon bond, where the A and B rings are connected in parallel (Fig. S13). This structural difference leads to different conversion rates between flavonoids and anthraquinones; conversion rates for all rhamnosylated flavonoids were less than 6%, while those for the anthraquinone derivatives were higher than 15%. ER and AR have hydroxylated or methylated functional groups in the C ring of anthraquinones (Fig. S13). However, there was no functional group in the C ring of QR. Thus, a functional group in the C ring of the ER and AR might have strictly hindered the active binding site of ThnM1.

**TABLE 1:** Mass analyses of different methylated anthraquinone and flavonoid products and their relative conversion rates determined from *in vitro* reactions

Substrate	Mass of substrate (amu)	Mass of product expected (amu)	Mass of product observed (amu)	Product yield (%)
Quinizarin-4-*O*-α-l-rhamnoside	386.1002	423.1056 [(M + Na^+^), *m/z*^+^]	423.1060 [(M + Na^+^), *m/z*^+^]	95
Astilbin	450.1162	465.1391 [(M + H^+^), *m/z*^+^]	465.1360 [(M + H^+^), *m/z*^+^]	<5
Hesperidin	610.1898	625.2127 [(M + H^+^), *m/z*^+^]	625.2130 [(M + H^+^), *m/z*^+^]	<5
Emodin-3-*O*-α-l-rhamnoside	416.1107	431.1337 [(M + H+), *m/z*^+^]	431.1331 [(M + H+), *m/z*^+^]	<15
Anthrarufin-5-*O*-α-l-rhamnoside	386.1002	423.1056 [(M + Na+), *m/z*^+^]	423.1044 [(M + Na+), *m/z*^+^]	<15
Diosmin	608.1741	623.1970 [(M + H+), *m/z*^+^]	623.1969 [(M + H+), *m/z*^+^]	<6

There are two potential approaches to generate methylated sugar-conjugated NPs. First, methylated nucleoside diphosphate (NDP) sugars are produced and eventually transferred to NPs by glycosyltransferase enzymes. The second approach involves the conjugation of a sugar molecule to a core metabolite, which is then modified by a sugar MT. To determine the possibility of the first approach and involvement of ThnM1, methylation assays with UDP-glucose, TDP-glucose, and TDP-rhamnose were performed, and the reaction mixtures were analyzed using HPLC and LC-MS. However, these reactions failed to yield the methylated derivatives of the NDP sugars (data not shown). This evidence demonstrates that ThnM1 follows a pathway in which the methyl group is transferred to a glycosylated NP rather than to an NDP-sugar (Fig. S14).

### Whole-cell biotransformation of quinizarin.

After biochemical characterization of ThnM1, the enzyme was applied to produce quinizarin-4-*O*-α-l-methylrhamnoside, from quinizarin, using a whole-cell biotransformation approach. E. coli strain S2 was generated by engineering E. coli BL21(DE3), which harbors a sugar transfer cassette and a sugar methylation cassette (2, 30). For biotransformation, the induced culture of E. coli strain S2 with 10% glucose was fed with 2 mM methionine and different concentrations (2 mM to 16 mM) of substrate. Among all concentrations of quinizarin tested, 4 mM was the optimal concentration of the substrate for the production of the methylated derivative, resulting in a conversion rate of ~95% (Table S4 and Fig. S15). At concentrations greater than 4 mM, the conversion rate dropped strikingly. The exogenously supplemented molecules exert various biological effects on cell’s metabolic pathways and overall metabolism. The higher concentration of quinizarin added to the cell could be toxic to E. coli and resulted in no conversion of quinizarin when supplemented above 10 mM.

To enhance the cytosolic content of SAM, the donor substrate of the methyl group of MT, engineered strains were generated and evaluated for the cytosolic titer of SAM (Table S5). Silencing *speD* or overexpressing *metK* resulted in enhanced SAM by 1.3-fold or 1.8-fold, respectively (Table S5). However, the combined approach of overexpressing *metK* and silencing *speD* significantly enhanced SAM by approximately 3.4-fold (Table S5). Thus, the integrated approach could have significantly improved the production of quinizarin-4-*O*-α-l-methylrhamnoside. However, other process development approaches such as development of high-cell-density-growing cells, improved fermentation conditions, fed-batch addition of substrate, etc., could further enhance the titer of the end product.

### Analysis and characterization of methylated derivative of quinizarin.

**FIG 4:**
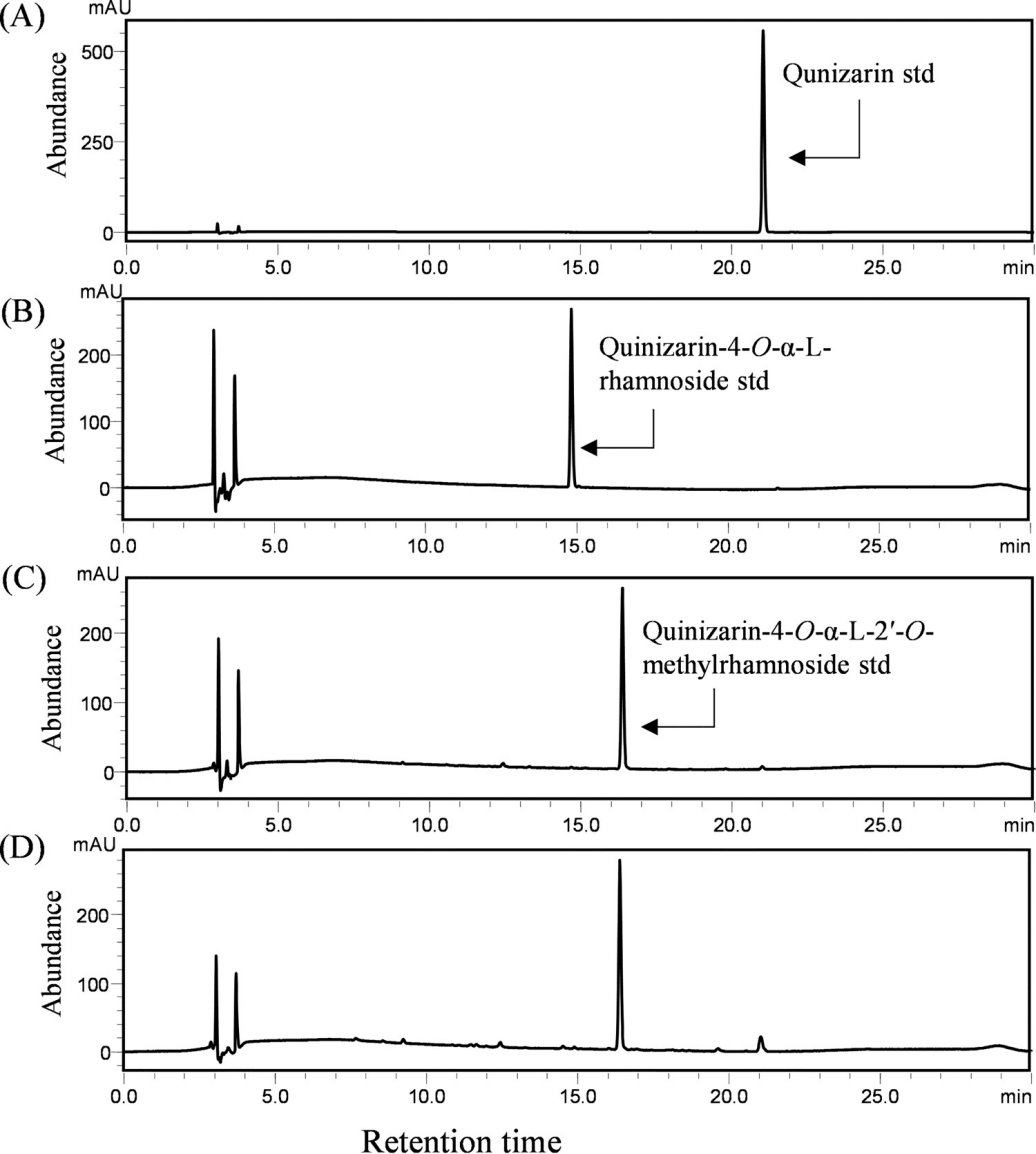
Whole-cell biotransformation of quinizarin to quinizarin-4-*O*-α-L-2′-*O*-methylrhamnoside by engineered *E. coli* S2 strain overexpressing anthraquinone glycosyltransferase, sugar-MT (ThnM1), TDP-rhamnose sugar biosynthetic pathway overexpressing plasmid, and SAM synthase overexpressing plasmid. HPLC-PDA chromatogram analyses of (A) quinizarin standard, (B) quinizarin-4-*O*-α-L-rhamnoside standard, (C) quinizarin4-*O*-α-L-2′-*O*-methylrhamnoside standard, and (D) the biotransformation reaction sample.

**FIG 5:**
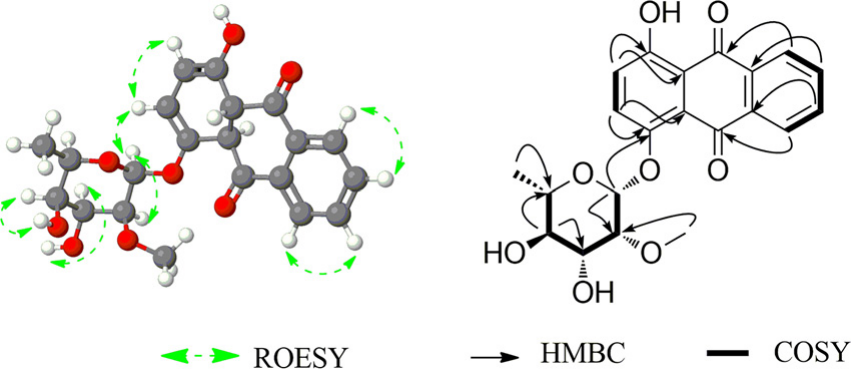
HMBC, COSY, and ROESY correlations of quinizarin-4-*O*-α-l-2′-*O*-methylrhamnoside.

For structural elucidation of the product, compounds were purified from crude extracts by preparative HPLC (prep-HPLC). The HPLC chromatogram of the quinizarin reaction revealed a peak corresponding to its standard retention time of 21.8 min and a new peak at a retention time of 16.4 min with UV absorbance at 420 nm (Fig. 4). The new peak was analyzed by ESI-tandem MS (ESI-MS/MS). The peak showed a total mass of (M+Na)^+^ *m/z* = 423.1056 in the positive ion mode (Fig. 2E), which resembled the methylated derivative of QR. The compound was purified, freeze-dried, and dissolved in 400 μL of dimethyl sulfoxide (DMSO)-*d6* for nuclear magnetic resonance (NMR) analyses including 1-D NMR (^1^H-NMR and ^13^C-NMR) and two-dimensional (2D) NMR (heteronuclear multiple bonded connectivity [HMBC], heteronuclear single quantum coherence [HSQC], correlation spectroscopy [COSY], and rotating frame Overhauser enhancement spectroscopy [ROESY]), as shown in Fig. S16 to S18, Fig. 5, and Table S6.

A Single –OCH_3_ spectrum was visible in both ^1^H and ^13^C NMR at 3.47 ppm (3H, s) and 59.35 ppm, respectively. Furthermore, 2D NMR analyses, such as ^1^H-^13^C HSQC, ^1^H-^13^C HMBC, ^1^H-^1^H COSY, and ^1^H-^1^H ROESY experiments, were performed to determine the exact position of methylation (Fig. 5). ^1^H-^13^C HSQC showed a cross-peak illustrating a correlation between protons (δ 3.47 ppm) and the carbon of the methoxy group (δ 59.35 ppm). Moreover, ^1^H-^13^C HMBC showed a cross-peak depicting correlations between C-2′ (δ 80.68 ppm) and protons of the methoxy group (δ 3.47 ppm). ^1^H-^1^HCOSY correlation was observed between the protons of C-1′ and C-2′ but absent in the methoxy groups (C-7′), suggesting the presence of more than one bond distance. Evidence from various NMR analyses revealed that the methylated derivative produced by E. coli S2 whole-cell biotransformation of quinizarin was quinizarin-4-*O*-α-l-2′-*O*-methylrhamnoside, a new compound.

### Cytotoxicity test.

Quinizarin and quinizarin-4-*O*-*α*-l-2-*O*-methylrhamnoside (QRM) were evaluated for their *in vitro* cytotoxicity using a 3-(4,5-dimethylthiazol-2-yl)-2,5-diphenyltetrazolium bromide (MTT) colorimetric assay against three different cancer cell lines. The results are shown in Fig. S19, where of the two anthraquinone derivatives tested, QRM exhibited the highest cytotoxicity. The 50% inhibitory concentration (IC_50_) values of QRM for the U87MG, SNU-1, and A375SM cell lines were 175.8, 98.78, and 177.9 μM, respectively (Table 2). Among the three different cancer cell lines tested, QRM showed higher cytotoxic activity, and the SNU-1 cell line was the most sensitive to QRM, followed by U87MG and A375SM. These results suggest that the conjugation of the methylated rhamnosyl moiety could positively influence the anticancer potential of quinizarin.

**TABLE 2:** Cell cytotoxicity assay of quinizarin and quinizarin-4-*O*-α-l-2′-*O*-methylrhamnoside against different cancer cell lines with IC_50_ values

IC_50_ (μM)	U87MG	SNU-1	A375SM
Quinizarin	>200	>200	200
Quinizarin-4-*O*-α-l-2′-*O*-methylrhamnoside	175.8	98.78	177.9

### Conclusions.

The identification of a regioselective MT is particularly interesting to produce chemically diverse molecules by alkylation using inexpensive approaches. ThnM1 has been previously reported to be a putative MT encoded by *thnM1* in the BGC of 1-(α-l-(2-*O*-methyl)-6-deoxymannopyranosyloxy)-3,6,8-trimethoxynaphthalene. In this study, *in vitro* enzyme reactions revealed that ThnM1 efficiently converted rhamnose-conjugated anthraquinones and flavonoids to their methylated forms by transferring a methyl group at the 2′-OH position of the rhamnose moiety. In addition, a whole-cell biotransformation platform was developed by incorporating a rhamnose biosynthetic cassette, anthraquinone rhamnosyltransferase, SAM synthase, and CRISPR-cas9-mediated silencing of SAM decarboxylase. This integrated metabolic engineering approach led to the highest production level of quinizarin-4-*O*-α-l-2′-*O*-methylrhamnoside for the first time in microbial cells. The newly synthesized quinizarin-4-*O*-α-l-2′-*O*-methylrhamnoside exhibited higher cytotoxicity than its parental compound, quinizarin, in three different cell lines, U87MG, SNU-1, and A375SM. In summary, functional characterization of ThnM1, which is involved in regiospecific methylation of rhamnose-containing NPs, opens possibilities for the synthesis of methoxyrhamnose-conjugated molecules using microbial cells by simple fermentation.

## MATERIALS AND METHODS

### Chemicals and reagents.

All culture media and components were purchased from BD Biosciences (Franklin Lakes, NJ, USA). High-performance liquid chromatography (HPLC)-grade acetonitrile, trifluoroacetic acid (TFA), and water were purchased from Mallinckrodt Baker (Phillipsburg, NJ, USA). Isopropyl-β-d-1-thiogalactoside (IPTG) was purchased from GeneChem, Inc. (Daejeon, Republic of Korea). SAM and flavonoids were purchased from Sigma-Aldrich (St. Louis, MO, USA). All chemicals were of high-grade quality and purchased from commercially available sources.

### Cloning of *thnM1*.

DNA isolation, digestion, ligation, and other DNA manipulations were performed using standard methods. Genomic DNA was isolated from the *Nocardia* sp. CS682 using a Qiagen DNeasy tissue kit (Qiagen, Gaithersburg, MD, USA) that was further used as a template for PCR amplification of the *thnM1* gene. For PCR, *Pfu* DNA polymerase was used under the following conditions: an initial denaturation at 95°C for 7 min; 30 cycles of denaturation at 92°C for 1 min, annealing at 58°C for 1 min, extension at 72°C for 1 min, and a final extension at 72°C for 7 min. The PCR products were purified from 0.6% agarose gel and ligated into pGEM-T Easy vector (Promega, Wisconsin, USA) using T4 DNA ligase (Promega). DNA manipulation was carried out in E. coli XL1-Blue (StrateGene). Recombinant plasmids were verified by sequencing. The pET-32a(+) expression vector was used for protein expression. All the strains, plasmids, and PCR primers used in this study are listed in Tables S1 and S2.

### Expression and purification of recombinant proteins.

The recombinant plasmid (pET32-ThnM1) was generated by cloning *thnM1* into the pET-32a(+). The recombinant plasmid harboring E. coli BL21(DE3) (StrateGene, La Jolla, CA, USA) and E. coli ThnM1 was used as an expression host for protein production. E. coli-ThnM1 was cultured and grown in Luria Bertani medium (LB) supplemented with 50 μg/mL ampicillin at 37°C with shaking at 180 rpm until the optical density at 600 nm (OD_600_) reached 0.8. The culture was then induced with IPTG at a final concentration of 0.4 mM and continued to grow at 20°C for 18 h. Cells were harvested by centrifugation (20 min, 824 × *g*) at 4°C, washed with 50 mM Tris-HCl buffer (pH 7.8) containing 100 mM NaCl, subsequently suspended in 1 mL of the same buffer, and lysed by sonication using a Sonosmasher (Ultrasonic, Ulsso Hitec, Republic of Korea.) in an ice bath. Cell debris were removed by centrifugation at 10,000 × *g* for 20 min at 4°C. The target protein was purified using the TALON cobalt resin kit for His-tag purification (TaKaRa Bio, Shiga, Japan). The fractions of the purified protein were analyzed by sodium dodecyl sulfate-polyacrylamide gel electrophoresis (SDS-PAGE), and the pure fractions were pooled using Amicon Ultra-15 centrifugal filters (Millipore; 30,000 nominal molecular weight limit [NMWL]). Protein concentration was determined using the Bradford method (31). The purified protein was stored in 10% glycerol containing 50 mM Tris-HCl (pH 7.8) buffer at −70°C for future use. For SDS-PAGE analysis, 12% (wt/vol) polyacrylamide was used as the separating gel. Protein bands were visualized using Coomassie brilliant blue R-250 (Tokyo Chemical Industry, Tokyo, Japan) staining.

### Characterization of methylation activity of ThnM1.

To determine the MT activity of ThnM1, an *in vitro* enzymatic reaction was carried out in a reaction mixture of 200 μL. The reaction mixture comprised 50 mM Tris-HCl buffer (pH 7.5), 2 mM SAM, 4 mM substrate, 2 mM MgCl_2_, and 50 μg ThnM1. The following substrates were used: flavanone glycoside (hesperidin, naringenin 7-*O*-β-d-glucoside, naringenin 4′-*O*-β-d-glucoside), flavanonol (astilbin), anthraquinones and glycosides (QR, emodin-3-*O*-α-l-rhamnoside [ER], anthrarufin-5-*O*-α-l-rhamnoside [AR], alizarin, emodin, anthrarufin, chrysazin/dantron, and quinizarin), flavonol (morin hydrate), flavone (diosmin), polyketide (nargenicin A), and naphthalene (1,2-dihydronaphthalene, 1,5-dihydronaphthalene, 1,6-dihydronaphthalene, 1,7-dihydronaphthalene, 1,8-dihydronaphthalene, 2,3-dihydronaphthalene, and 2,7-dihydronaphthalene). Reaction mixtures were incubated at 40°C for 3 h and quenched by adding a double volume of chilled methanol. Precipitated proteins were removed by centrifugation at 10,000 × *g* for 30 min at 4°C. The supernatant was collected for product analysis using HPLC and liquid chromatography-mass spectrometry (LC-MS).

### Effects of temperature, buffers, cofactors, and pH.

The temperature profile for ThnM1 between 4 and 75°C was obtained with Tris-HCl buffer (pH 7.5), 2 mM SAM, 2 mM MgCl_2,_ 50 μg ThnM1, and 2 mM QR for 1 h. The product was quantified by HPLC. Similarly, the effect of pH was investigated using 50 mM different buffers, namely, carbonate-bicarbonate buffer, citrate buffer, phosphate buffer, and Tris-HCl buffer, having pH ranging from 4.0 to 10.5 at 40°C. To determine the metal ion dependence of ThnM1, methylation of 2 mM QR was carried out at 40°C and pH 7.5 in the presence of different metal ions (2 mM), such as Mg^2+^, Mn^2+^, Ca^2+^, Cu^2+^, Fe^2+^, Fe^3+^, Zn^2+^, Ni^2+^, Co^2+^, and EDTA (a negative control).

### Enzyme kinetics of ThnM1.

The Bradford assay was performed to measure the protein concentration. Bovine serum albumin (BSA) was used to prepare a six-point reference calibration curve in the range of 0.0 to 100 μg/mL. Bradford reagent was added to the samples, and the absorbance was measured at 595 nm after 20 min. The standard curve was prepared in duplicates, and the samples were prepared in triplicates. To determine the kinetic parameters of ThnM1 with QR, a reaction mixture containing ThnM1 (2 μg), SAM (2 mM), MgCl_2_ (2 mM), Tris-HCl buffer (50 mM, pH 7.5), and various concentrations of QR (5 to 200 μM) was prepared in a total volume of 200 μL. Similarly, to determine the ThnM1 kinetic parameters for SAM, a 200-μL reaction mixture containing ThnM1 (2 μg), QR (100 μM), MgCl_2_ (2 mM), Tris-HCl buffer (50 mM, pH 7.5), and various concentrations of SAM (5 to 200 μM) was prepared. ThnM1 assays with different enzyme concentrations were carried out in a 200 μL reaction mixture containing ThnM1 (0.5 to 5 μg), 5 μM QR, 2 mM SAM, 2 mM MgCl_2_, and Tris-HCl buffer (50 mM, pH 7.5) for 30 min. Time-dependent *in vitro* conversion of QR to quinizarin-4-*O*-α-l-2-*O*-methylrhamnoside (QRM) was measured every 5 min for 30 min using 2 μg ThnM1, 5 μM QR, 2 mM SAM, 2 mM MgCl_2_, and Tris-HCl buffer (50 mM, pH 7.5). All reactions were terminated by adding 400 μL (double volume) of chilled methanol. After centrifugation (10,000 × *g* for 30 min), the supernatant was analyzed using HPLC. Data were obtained from triplicate experiments. Data are reported as the mean ± standard deviation. *V*_max_ and Michaelis-Menten constant, *K*_m_, values were determined using nonlinear regression analysis, assuming Michaelis-Menten steady-state kinetics (32).

### Generation of recombinant strains.

To produce methoxy-rhamnosylated derivatives using engineered E. coli, anthraquinone rhamnosyltransferase (7665) (33) from Saccharothrix espanaensis was cloned into pET-32a(+). E. coli S2 strain was generated by transforming pET32a(+)-7665(Am) CRISPRi-S1(Cm^r^) and pCDFDuet-metK-thnM1(Sm) into an E. coli strain harboring the rhamnose cassette piBR181-tgs.dh.ep.kr.pgm2 glf glk (Km) (30). It was subsequently used to produce QRM from quinizarin.

A two-way approach was employed to enhance the pool of SAM (the methyl group donor). The SAM titer was enhanced by overexpressing SAM synthase (*metK*) while repressing the expression of an enzyme responsible for the degradation of SAM (*speD*). *The metK* gene from E. coli K-12 was amplified from the E. coli genome using the primers listed in Table S2 and cloned into the NdeI and XhoI sites of pCDFDuet-1 to generate pCDFDuet-metK. Similarly, *thnM1* was cloned into the BamHI and HindIII sites of pCDFDuet-metK to generate pCDFDuet-metK-thnM1. Plasmids used for CRISPRi/dCas9-mediated transcriptional repression were obtained from Addgene (plasmid no. 65006). The silencing construct was generated as previously described (2). The specific targeting spacer of *speD* from the E. coli K-12 genomic DNA was identified in the untranslated region of *speD* to prevent RNA polymerase binding and elongation. Primer pair speRNA1-Fw/speRNA1-Rv was used to construct CRISPRi-S1. The primers were synthesized, phosphorylated with T_4_ polynucleotide kinase, and annealed. The inserts were then ligated into the BsaI-digested, dephosphorylated, gel-purified CRISPRi plasmid backbone. E. coli DH5α was used for cloning experiments. All CRISPRi plasmid arrays possessing synthetic-specific targeting spacers were verified by colony PCR with primer pair cPCR-Fw/cPCR-Rv (Table S2) followed by sequencing. Silencing constructs were then transformed into E. coli BL21(DE3) cells expressing the cassette for rhamnosylation, methylation, and SAM synthase. The silencing of *speD* and overexpression of *metK* were evaluated by reverse transcriptase PCR (RT-PCR) analysis.

### Whole-cell biotransformation.

E. coli S2 was inoculated into 5 mL of LB broth containing an appropriate amount of antibiotics (50 μg/mL ampicillin, 50 μg/mL chloramphenicol, 50 μg/mL streptomycin, and 50 μg/mL kanamycin) when needed. Cells were grown at 37°C overnight. Next, 200 μL of the bacterial culture was aseptically transferred to 250-mL flasks containing 50 mL of LB and the same set of antibiotics. They were allowed to grow at 37°C until the optical density at 600 nm (OD_600_) reached 0.8. Cells were then induced with 0.4 mM IPTG at 20°C for 18 h for protein expression. Quinizarin was dissolved in dimethyl sulfoxide (DMSO) and added to all flasks at a final concentration of 300 μM for biotransformation. After incubation at 20°C for 48 h, all cultures were extracted with double volume of ethyl acetate. The organic supernatant fraction was then concentrated using a rotary evaporator. The concentrated sample was dissolved in an appropriate volume of methanol for HPLC analysis (to be carried out at 420 nm).

### Analytical method: HPLC.

Reverse-phase HPLC analyses were performed using a C_18_ column (Mightysil RP-18 GP 250-4.6, 5 μm) at a UV absorbance of 420 nm. The mobile phase consisted of solvent A (water with 0.1% trifluoroacetic acid [TFA]) and solvent B (acetonitrile). The program for HPLC was as follows: 10% acetonitrile at 0 min, 30% acetonitrile at 5 min, 50% acetonitrile at 10 min, 90% acetonitrile at 15 min, 70% acetonitrile at 18 min, and 10% acetonitrile at 30 min, with a flow rate of 1 mL/min. High-resolution quadrupole time of flight electrospray ionization-mass spectrometry (HR-QTOF ESI/MS) analysis was performed in the positive ion mode using an Acquity column (UPLC; Waters Corp., Billerica, MA, USA) coupled with a SYNAPT G2-S column (Waters Corp.).

### Purification and characterization of methoxy derivatives.

The purification of methoxy derivatives generated in this study was performed by preparative HPLC (prep-HPLC) equipped with a C_18_ column (YMC-Pack ODS-AQ; 250 by 20 mm inside diameter [i.d.], S-10 μm) connected to a UV detector (420 nm) using a 40-min binary program with different concentrations of acetonitrile—10% (0 to 5 min), 40% (5 to 10 min), 60% (10 to 15 min), 70% (15 to 25 min), 90% (25 to 35 min), and 10% (35 to 40 min)—at a flow rate of 10 mL/min. The collected fractions from prep-HPLC were reanalyzed using analytical HPLC to determine their purity. Highly pure fractions were pooled, dried, and lyophilized to remove water or moisture. Further, the fully dried pure compound was dissolved in DMSO-*d6* and subjected to NMR analysis (700 MHz). One-dimensional NMRs (^1^H NMR and ^13^C NMR) and 2-D NMRs (heteronuclear multiple quantum coherence [HMQC], rotating frame Overhauser enhancement spectroscopy [ROESY], and heteronuclear multiple bonded connectivity [HMBC]) were used, as needed, to elucidate the structure of the compound.

### Assessment of cytotoxicity.

SNU-1 (gastric cancer cells) was maintained in RPMI 1640 medium supplemented with 10% fetal bovine serum (FBS) (Invitrogen, USA). U87MG (glioblastoma) cells were grown in a minimum essential medium supplemented with 10% FBS (Gibco). A375SM cells were grown in Dulbecco’s modified Eagle’s medium supplemented with 10% FBS. All cells were maintained at 37°C in a humidified 5% CO_2_ incubator. Cancer cells were plated in 96-well culture plates at a density of 2 × 10^3^ cells/well and then treated with various concentrations of quinizarin and QRM for 72 h. Cell growth was measured using the 3-(4,5-dimethylthiazol-2-yl)-2,5-diphenyltetrazolium bromide (MTT) colorimetric assay.

### Compliance with ethical standards.

The article does not contain any studies with human participants or animals performed by any of the authors.
