# Supplementary material for: Functional Characterization of a Regiospecific Sugar-O-Methyltransferase from Nocardia
Source: Appl Environ Microbiol. 2022 Jun 15;88(13):e00754-22. doi: 10.1128/aem.00754-22 (PMC9275233; doi:10.1128/aem.00754-22)
Supplement: Supplemental file 1 — Tables S1 to S6 and Fig. S1 to S19. Download aem.00754-22-s0001.pdf, PDF file, 3.3 MB [file aem.00754-22-s0001.pdf]

Supplementary data of

Functional characterization of a regiospecific sugar-*O*-methyltransferase from *Nocardia*

Purna Bahadur Poudel[1*], Ramesh Prasad Pandey[1*], Dipesh Dhakal[1], Tae-Su Kim [1], Trang Thi Huyen Nguyen[1], Hye Jin Jung[1,2], Hee Jeong Shin[1], Binod Timalsina[3], and Jae Kyung Sohng[1,2, * *]

[1]*Institute of Biomolecule Reconstruction (iBR), Department of Life Science and Biochemical Engineering, Sun Moon University, 70 Sun Moon-ro 221, Tangjeong-myeon, Asan-si, Chungnam 31460, Korea.*

[2]*Department of Biotechnology and Pharmaceutical Engineering, Sun Moon University, 70 Sun Moon-ro 221, Tangjeong-myeon, Asan-si, Chungnam 31460, Korea.*

[3]*Department of Anatomy, Dongguk University College of Medicine, Gyeongju 38066, Korea.*

[*]These authors are equally contributed.

**Corresponding author: Prof. Jae Kyung Sohng

Tel: +82 (41) 530-2246

Fax: +82 (41) 530-8229

Email: sohng@sunmoon.ac.kr

Table and figure of contents

Table S1. List of strains and plasmids used in this study for heterologous expression and constructed for mutant strain. 6

Table S2. List of primers used in this study for cloning gene and constructed for mutant strain. . 7

Table S3. The kinetic parameter of ThnM1 with quinizarin-4-*O*-*α*-L-rhamnoside and *S*-adenosyl-L-methionine 8

Table S4. Different concentrations of substrate (quinizarin) for the bioconversion in *E. coli* S2.. 9

Table S5. Effects of SAM concentration in the mutant strain (*E. coli*-SpeD, *E. coli*-MetK, *E. coli*-Metk/SpeD, *E. coli*-S2) compared with wild type strain.. 10

Table S6. Comparison of ^{1}H- and^{13}C-NMR chemical shifts of quinizarin, quinizarin-4-*O*-*α*-L-rhamnoside, and quinizarin-4-*O*-*α*-L-2′-*O*-methylrhamnoside measured in DMSO-*d6* solvent....... 11

Figure S1. 12

Evolutionary relationship of a methyl transferase family proteins from different sources. The name of the enzyme, strain and accession number of the amino acid sequence is shown in the tree. Evolutionary analyses were conducted in MEGA X software (3). The evolutionary history was inferred by using the Maximum Likelihood method and JTT matrix-based model(4). A total of 133 amino acid sequences were used for the phylogenetic tree analysis. Initial tree(s) for the heuristic search were obtained automatically by applying Neighbor-Join and BioNJ algorithms to a matrix of pairwise distances estimated using the JTT model, and then selecting the topology with superior log likelihood value. The branch length indicates the evolutionary distance between different enzymes. All positions containing gaps and missing data were eliminated. The significance was tested by bootstrap test (1000 replicates) using MEGA X. *Nocardia* sp.CS682 (ThnM1 WP_135236660.1) is shown in the tringle symbols. 12

Figure S2. 13

Homologous amino acid sequences alignment of ThnM1with selected previously characterized methyltransferase. Sequences alignment of ThnM1 with other *O*-methyltransferases such as natural product sugar methyltransferase MycE in mycinamicin pathway from *Micromonospora griseorubida* (3SSM_D),) ElmM1 from *Streptomyces olivaceus* (Q9AJU2.1), ElmM2 from *Streptomyces olivaceus* (Q9AJU2.1), OleY from *Streptomyces antibioticus* (O87833.1), GerMIII from *Streptomyces sp*. KCTC 0041BP (AY118081), Biki from *Streptomyces bikiniensis* (AAS79456.1), TylE *Streptomyces fradiae* (Q9ZHQ4.1) and BusK from Saccharopolyspora pogona (AAY88928.1) The red box is the active site domain of *O*-methyltransferases. Their amino acid numbering is based on the crystal structure of MycE (PDB number: 3SSM_A). 13

Figure S3. 14

12% SDS-PAGE analysis of heterologously overexpressed ThnM1 protein in *E. coli* BL21 (DE3) expression host. Lane 1: clear lysate of ThnM1; Lane 2: unclear lysate of ThnM1; Lane 3: Insoluble fraction of ThnM1; Lane 4: Purified ThnM1; M: Standard protein ladder.......... 14

Figure S4. 15

Structures of different substrate that are not accepted by ThnM1 in *in vitro* reaction............. 15

Figure S5. 16

A) Reaction scheme of methylation of emodin 3-*O*-α-L-rhamnoside by ThnM1 in the presence of SAM at 40ºC for 3 h. 16

B) (i) HPLC-PDA chromatogram of reaction mixture of emodin 3-*O*-*α*-L-rhamnoside with ThnM1 (ii) UV/VIS of methylated product of emodin 3-*O*-*α*-L-rhamnoside.......... 16

C) (iii) HPLC-PDA chromatogram and (iv) UV/VIS of emodin 3-*O*-*α*-L-rhamnoside Std..... 16

D) HR-QTOF ESI/MS analysis of methylated product of emodin 3-*O*-*α*-L-rhamnoside 16

Figure S6. 17

A) Reaction scheme of methylation of anthrarufin-5-*O*-*α*-L-rhamnoside by ThnM1 in the presence of SAM at 40ºC for 3 h. 17

B) (i) HPLC-PDA chromatogram of reaction mixture of anthrarufin-5-*O*-*α*-L-rhamnoside with ThnM1 (ii) UV/VIS of methylated product of anthrarufin-5-*O*-*α*-L-rhamnoside. 17

C) (iii) HPLC-PDA chromatogram and (iv) UV/VIS of anthrarufin-5-*O*-*α*-L-rhamnoside Std. 17

D) HR-QTOF ESI/MS analysis of methylated product of anthrarufin-5-*O*-*α*-L-rhamnoside. . 17

Figure S7. 18

A) Reaction scheme of methylation of astilbin by ThnM1 in the presence of SAM at 40ºC for 3 h. 18

B) (i) HPLC-PDA chromatogram of reaction mixture of astilbin with ThnM1 (ii) UV/VIS of methylated product of astilbin. 18

C) (iii) HPLC-PDA chromatogram and (iv) UV/VIS of astilbin Std. 18

D) HR-QTOF ESI/MS analysis of methylated product of astilbin. 18

Figure S8. 19

A) Reaction scheme of methylation of hesperidin by ThnM1 in the presence of SAM at 40ºC for 3 h. 19

B) (i) HPLC-PDA chromatogram of reaction mixture of hesperidin with ThnM1 (ii) UV/VIS of methylated product of hesperidin. 19

C) (iii) HPLC-PDA chromatogram and (iv) UV/VIS of hesperidin Std. 19

D) HR-QTOF ESI/MS analysis of methylated product of hesperidin 19

Figure S9. 20

A) Reaction scheme of methylation of diosmin by ThnM1 in the presence of SAM at 40ºC for 3 h. 20
B) (i) HPLC-PDA chromatogram of reaction mixture of diosmin with ThnM1 (ii) UV/VIS of methylated product of diosmin C) (iii) HPLC-PDA chromatogram and (iv) UV/VIS of diosmin Std 20
D) HR-QTOF ESI/MS analysis of methylated product of diosmin. 20
Figure S10. 21
Percentage conversion of all different substrates used in this study. The percent conversion was calculated by dividing the integrated peak area of a product by the sum of integrated peak area of product and substrate. 21
Figure. S11 22
Probing of ThnM1 assay conditions and determination of conditions for measuring initial velocity. A) Effect of different temperatures on the activity of purified ThnM1. B) Effect of pH on the activity of purified ThnM1. C) Different metal ions on the activity of purified ThnM1. D) ThnM1 assays with different enzyme concentrations. Assays comprising QR (5 μM), SAM (2 mM), $MgCl_2$ (2 mM) were performed in Tris-HCl buffer (50 mM, pH 7.5) with varying [ThnM1] (0.5-5 μg) at 40°C for 30 min. E) A time course of ThnM1 assay comprising QR (5 μM), ThnM1 (2 μg), SAM (2 mM), $MgCl_2$ (2 mM) were performed in Tris-HCl buffer (50 mM, pH 7.5) at 40°C within 30 min. 22
Figure S12. 23
Proposed reaction mechanism of MycE in natural product sugar methoxide biosynthesis [5]. 23
Figure S13. 24
Ring structure of flavonoid and anthraquinone. 24
Figure S14. 25
Two possible approaches for the generation of 1-(α-L-(2-*O*-methyl)- 6-deoxymanno-pyranosyloxy)-3,6,8- trimethoxy naphthalene from THN using TDP-rhamnose and ThnM1. The pathway in blue arrow shows the generation of methylated NDP-sugar and eventual transfer of sugar moiety to aglycone by a glycosyltransferase enzyme. The pathway in black arrows shows the conjugation of a sugar molecule to a core metabolite followed by modification of sugar by a sugar methyltransferase 25
Figure S15. 26
HPLC-PDA analyses of whole cell bioconversion reaction mixture of quinizarin to quinizarin-4-*O*-*α*-L-methylrhamnoside by feeding different concentration of substrate. (i) 2mM quinizarin, (ii) 4mM quinizarin, (iii) 8mM quinizarin, (iv) 10mM quinizarin, (v) 12mM quinizarin, and (vi) 16mM quinizarin 26
Figure S16. 27
(a) ^{1}H NMR spectrum of quinizarin-4-*O*-*α*-L-2′-*O*-methylrhamnoside at 700MHz in DMSO-d_6 27

(b) ^{13}C NMR spectrum of quinizarin-4-*O*-*α*-L-2′-*O*-methylrhamnoside at 176 MHz in DMSO-d_6 28
(c) ^1H-^1H COSY NMR of quinizarin-4-*O*-*α*-L-2′-*O*-methylrhamnoside 29
(d) ^1H-^1H ROSEY NMR of quinizarin-4-*O*-*α*-L-2′-*O*-methylrhamnoside 30
(e) ^1H-^{13}C HSQC-DEPT NMR of quinizarin-4-*O*-*α*-L-2′-*O*-methylrhamnoside 31
(f) ^1H-^{13}C HMBC NMR of quinizarin-4-*O*-*α*-L-2′-*O*-methylrhamnoside. 32
Figure S17. 33
(a) ^1H NMR spectrum of quinizarin-4-*O*-*α*-L-rhamnoside at 800MHz in DMSO-d_6 33
(b) ^{13}C NMR spectrum quinizarin-4-*O*-*α*-L-rhamnoside in 176MHz, DMSO-d6. 34
(c) ^1H-^1H COSY NMR of quinizarin-4-*O*-*α*-L-rhamnoside. 35
(d) ^1H-^{13}C HSQC-DEPT NMR of quinizarin-4-*O*-*α*-L-rhamnoside. 36
(e) ^1H-^{13}C HMBC NMR of quinizarin-4-*O*-*α*-L-rhamnoside. 37
(f) DEPT 45 NMR spectrum of quinizarin-4-*O*-*α*-L-rhamnoside. 38
(g) DEPT 90 NMR spectrum of quinizarin-4-*O*-*α*-L-rhamnoside. 39
(h) DEPT 135 NMR spectrum of quinizarin-4-*O*-*α*-L-rhamnoside. 40
Figure S18. 41
(a) ^1H NMR spectrum of quinizarin at 300MHz in DMSO-d_6. 41
(b) ^{13}C NMR spectrum of quinizarin-4-*O*-*α*-L-rhamnoside at 176 MHz in DMSO-d_6. 42
Figure S19. 43
Cell cytotoxicity assay of quinizarin and quinizarin-4-*O*-*α*-L-2′-*O*-methylrhamnoside. Cells were treated with various concentrations (0.0 ~ 200μM) of each compound. 43
References 44

Table S1. List of strains and plasmids used in this study for heterologous expression and constructed for mutant strain.

Vectors/ strains	Description	Source/reference
pGEM-T®-easy vector	*E. coli* general cloning vector, Amp^r	Promega, Madison, WI, USA
pET32a(+)	Single T7 promotor, pBR322 *ori*, Km^r	Novagen
pET32-*thnM1*	pET32a(+) containing *thnM1* methyltransferase	This study
pET32a(+)-7665	pET32a(+) containing 7665 rhamnosyltransferase	Nguyen *et al* (1)
CRISPRi-*speD*	Vector containing cassette for silencing of *speD*	This study
pCDFDuet-*metK-thnM1*	pCDFDuet vector containing overexpression of SAM synthase (*metK*) and methyltransferase (*thnM1*)	This study
piBR181-*tgs.dh.ep.kr.pgm2.glf.glk*	TDP- rhamnose sugar biosynthetic gene cassette	Parajuli *et al* (2)
E. coli XL1Blue	*Δ(mcrA)183 Δ(mcrCB-hsdSMR-mrr)173 endA1 supE44 thi-1 recA1 gyrA1 gyrA96 relA1 lac*	Stratagene
E. coli BL21(DE3)	B; F- *omp*T *hsd*SB (*rB-mB-*) *gal dcm* (DE3)	Invitrogen
E. coli-thnM1	*E. coli* containing pET32-*thnM1*	This study
E. coli-speD	*E. coli* containing CRISPRi-*SpeD*	This study
E. coli-metK-thnM1	*E. coli* containing pCDFDuet-*metK-thnM1*	This study
E. coli S2	Final production host containing pET32a (+)-7665, CRISPRi-*speD*, pCDFDuet-*metK-thnM1* and piBR181-*tgs.dh.ep.kr.pgm2.glf.glk*	This study

Table S2. List of primers used in this study for cloning gene and constructed for mutant strain.

Vectors/ strains	Description	Comments
thnM1-F	GGATCCATGACCGGCAATGAAATGTA	Forward primer for amplification of *thnM1*
thnM1-R	AAGCTTTTAGCCGCCCACTGCAGCTT	Reverse primer for amplification of *thnM1*
metK-F	TCCATATGGCAAAACACCTTTTTACG	Forward primer for amplification of *thnM1*
metK-R	CCCTCGAGTTACTTCAGACCGGCAGCA	Reverse primer for amplification of *thnM1*
crRNA-*speD*-F	CCCATGCAAAAGTCATGATACCGCCGTA ATAG	Coding strand for CRISPR interference plasmid for silencing of *speD*
crRNA-*speD*-R	CTATTACGGCGGTATCATGACTTTTGCAT GGG	Non-coding strand for interference plasmid for silencing of *speD*

Table S3. The kinetic parameter of ThnM1 with quinizarin-4-*O*-*α*-L-rhamnoside and *S*-adenosyl-L-methionine

Substrate	K_m (μM)	V_{max} (μM $min^{-1}$$μg^{-1}$)
Quinizarin-4-*O*-*α*-L-rhamnoside	11.70 ± 1.97	0.0631 ± 0.0029
S-adenosyl-L-methionine	32.7 ± 7.06	0.0223 ± 0.0018

Table S4. Different concentrations of substrate (quinizarin) for the bioconversion in *E. coli* S2.

Substrate concentration (mM)	Quinizarin-4-*O*-α-L-methylrhamnoside
2	55%
4	95%
8	45%
10	15%
12	0%
16	0%

Table S5. Effects of SAM concentration in the mutant strain (*E. coli*-SpeD, *E. coli*-MetK, *E. coli*-Metk/SpeD, *E. coli*-S2) compared with wild type strain.

Strain	SAM (μM)	Relative SAM concentration (fold)
Wild	0.84 ± 0.04	1
E. coli-SpeD	1.09 ± 0.04	1.3
E. coli-MetK	1.51 ± 0.16	1.8
E. coli-Metk/SpeD	2.86 ± 0.24	3.4
E. coli-S2	2.64 ± 0.18	3.2

Table S6. Comparison of ^{1}H- and^{13}C-NMR chemical shifts of quinizarin, quinizarin-4-*O*-*α*-L-rhamnoside, and quinizarin-4-*O*-*α*-L-2′-*O*-methylrhamnoside measured in DMSO-*d6* solvent.

**Coupling constant is represented as J, whereas multiplicities are indicated by s (singlet), d(doublet), t (triplet), q (quartet), and m(multiplet), the*

^{1}H-NMR (700 MHz, DMSO-*d6*)				^{13}C-NMR (176 MHz, DMSO-*d6*)		
Position	Quinizarin	Quinizarin-4-*O*-*α*-L-rhamnoside	Quinizarin-4-*O*-*α*-L-2′-*O*-methylrhamnoside	Quinizarin	Quinizarin-4-*O*-*α*-L-rhamnoside	Quinizarin-4-*O*-*α*-L-2′-*O*-methylrhamnoside
1-OH	12.72 (*s*,1H)			156.71	150.01	150.38
2	7.45 (*s*,1H)	7.37 (d, *J* = 9.3 Hz, 1H)	7.69 (d, *J* = 7.0 Hz, 1H)	129.38	126.73	129.99
3	7.45 (*s*,1H)	7.65 (d, *J* = 9.4 Hz, 1H)	7.41 (d, *J* = 7.0 Hz, 1H)	129.38	129.01	126.19
4-OH	12.72 (*s*,1H)	12.88 (*s*,1H)	12.90 (s, 1H)	156.71	157.42	158.07
4a				112.71	115.54	116.09
5	7.99 (*dd*, *J* = 5.79, 3.31 Hz, 1H)	7.88 (d, *J* = 7.4 Hz, 1H)	8.22 (d, *J* = 7.0 Hz, 1H)	126.71	125.74	126.60
6	8.28 (*dd*, *J* = 5.81, 3.3 Hz, 1H)	8.15 (dd, *J* = 34.9, 7.5 Hz, 2H)	7.91 (m, 1H)	132.92	135.13	134.33
7	8.28 (*dd*, *J* = 5.81, 3.3 Hz, 1H)	8.15 (dd, *J* = 34.9, 7.5 Hz, 2H)	7.94 (m, 1H)	132.92	133.77	135.66
8	7.99 (*dd*, *J* = 5.79, 3.31 Hz, 1H)	7.91 (d, *J* = 7.3 Hz, 1H)	8.16 (d, *J* = 7.0 Hz, 1H)	126.71	126.07	127.25
8a				135.12	134.40	134.90
9				186.72	180.48	181.17
9a				112.71	120.12	120.92
10				186.72	188.39	188.90
10a				135.12	131.84	132.36
1’		5.48 (d, *J* = 1.7 Hz, 1H)	5.66 (s, 1H)		99.54	97.20
2’		5.08 (d, *J* = 3.6 Hz, 1H),	3.75 (dd, *J* = 7.0 Hz, 1H),		71.70	80.68
3’		4.91 (d, *J* = 4.9 Hz, 1H)	4.02 (m, 1H)		74.58	70.54
4’		4.80 (d, *J* = 4.9 Hz, 1H)	3.27 (td, 1H)		69.79	80.68
5’		4.05 (*s*,1H)	3.56 (dq,1H)		70.07	70.46
6’-CH_3		1.11 (*s*,2H)	1.10 (d, *J* = 7.0 Hz, 3H)		17.84	18.29
7-O-CH_3			3.47 (s, 3H)			59.35

chemical shift values are in ppm.

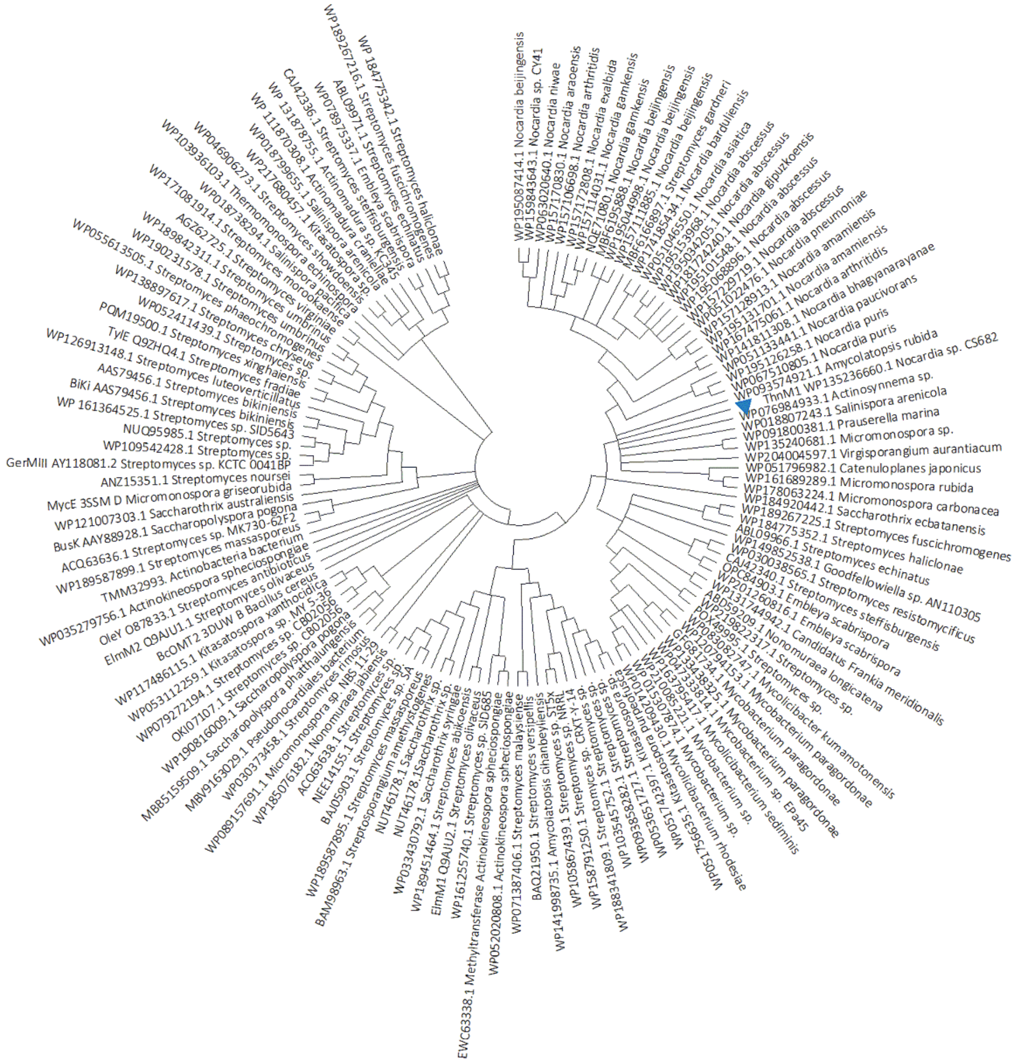

Figure S1.

Evolutionary relationship of a methyl transferase family proteins from different sources. The name of the enzyme, strain and accession number of the amino acid sequence is shown in the tree. Evolutionary analyses were conducted in MEGA X software (3). The evolutionary history was inferred by using the Maximum Likelihood method and JTT matrix-based model(4). A total of 133 amino acid sequences were used for the phylogenetic tree analysis. Initial tree(s) for the heuristic search were obtained automatically by applying Neighbor-Join and BioNJ algorithms to a matrix of pairwise distances estimated using the JTT model, and then selecting the topology with superior log likelihood value. The branch length indicates the evolutionary distance between different enzymes. All positions containing gaps and missing data were eliminated. The significance was tested by bootstrap test (1000 replicates) using MEGA X. *Nocardia* sp.CS682 (ThnM1 WP_135236660.1) is shown in the tringle symbols.

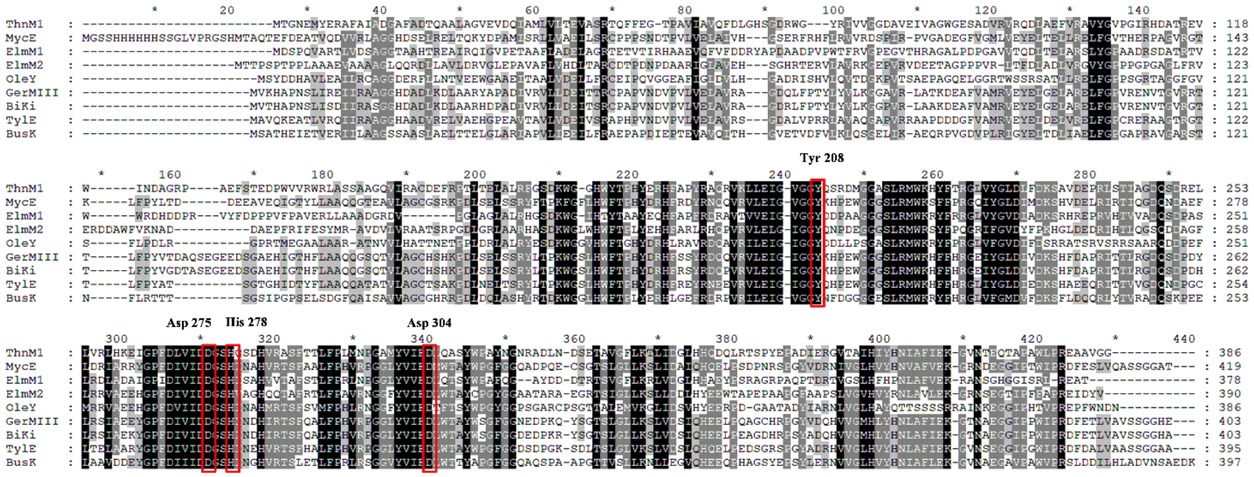

Figure S2.

Homologous amino acid sequences alignment of ThnM1with selected previously characterized methyltransferase. Sequences alignment of ThnM1 with other *O*-methyltransferases such as natural product sugar methyltransferase MycE in mycinamicin pathway from *Micromonospora griseorubida* (3SSM_D),) ElmM1 from *Streptomyces olivaceus* (Q9AJU2.1), ElmM2 from *Streptomyces olivaceus* (Q9AJU2.1), OleY from *Streptomyces antibioticus* (O87833.1), GerMIII from *Streptomyces sp*. KCTC 0041BP (AY118081), Biki from *Streptomyces bikiniensis* (AAS79456.1), TylE *Streptomyces fradiae* (Q9ZHQ4.1) and BusK from Saccharopolyspora pogona (AAY88928.1) The red box is the active site domain of *O*-methyltransferases. Their amino acid numbering is based on the crystal structure of MycE (PDB number: 3SSM_A).

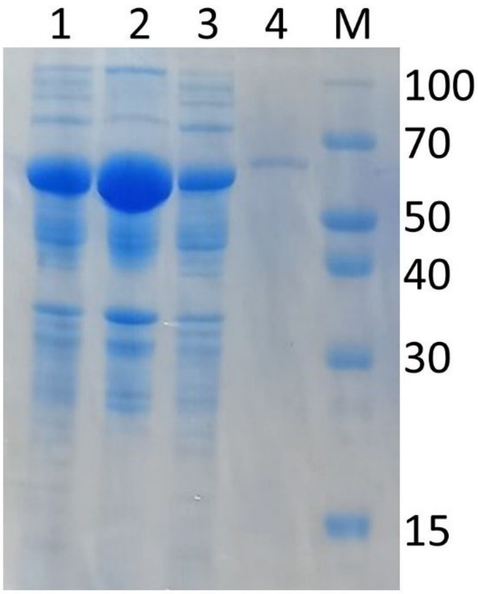

Figure S3.

12% SDS-PAGE analysis of heterologously overexpressed ThnM1 protein in *E. coli* BL21 (DE3) expression host. Lane 1: clear lysate of ThnM1; Lane 2: unclear lysate of ThnM1; Lane 3: Insoluble fraction of ThnM1; Lane 4: Purified ThnM1; M: Standard protein ladder.

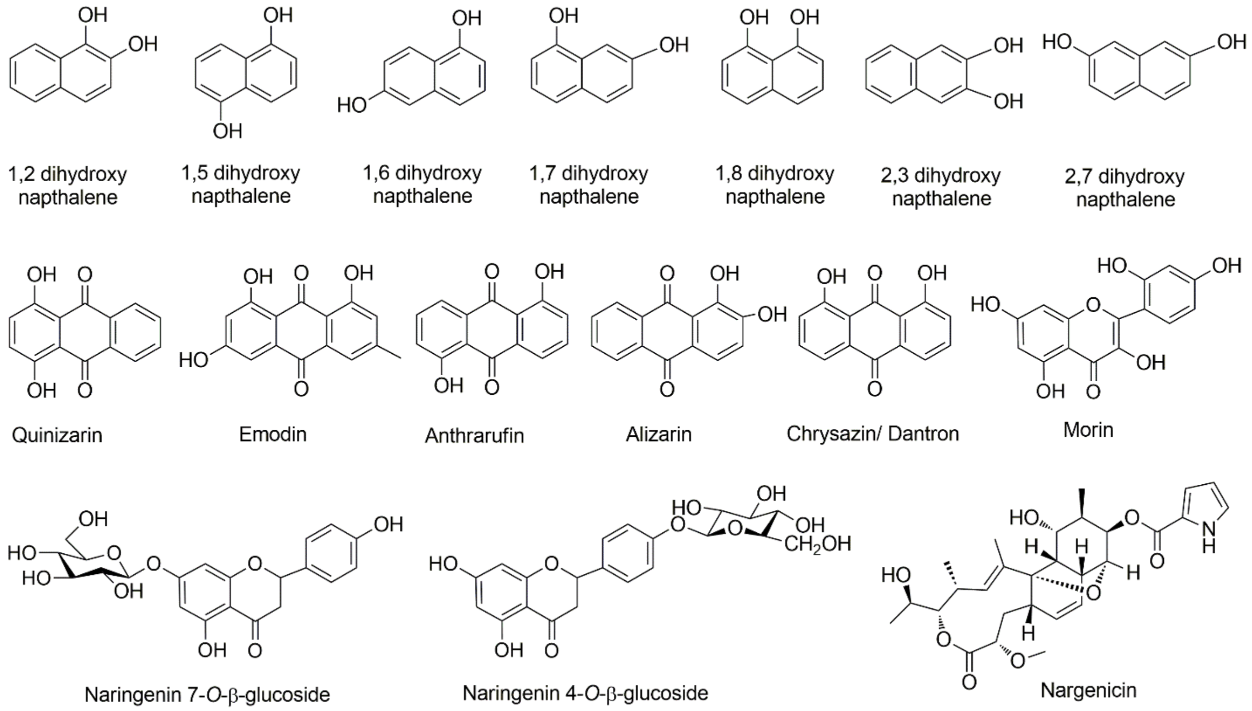

Figure S4.

Structures of different substrate that are not accepted by ThnM1 in *in vitro* reaction.

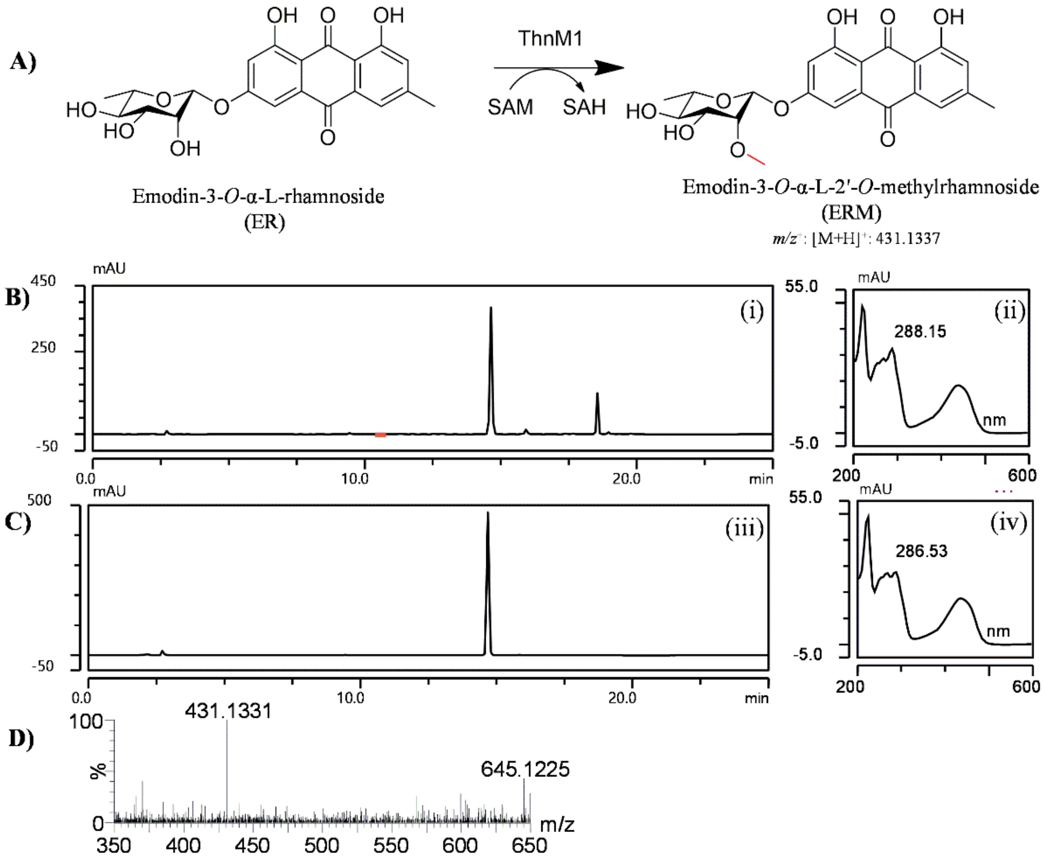

Figure S5.

A) Reaction scheme of methylation of emodin 3-*O*-α-L-rhamnoside by ThnM1 in the presence of SAM at 40ºC for 3 h.

B) (i) HPLC-PDA chromatogram of reaction mixture of emodin 3-*O*-*α*-L-rhamnoside with ThnM1 (ii) UV/VIS of methylated product of emodin 3-*O*-*α*-L-rhamnoside.

C) (iii) HPLC-PDA chromatogram and (iv) UV/VIS of emodin 3-*O*-*α*-L-rhamnoside Std.

D) HR-QTOF ESI/MS analysis of methylated product of emodin 3-*O*-*α*-L-rhamnoside

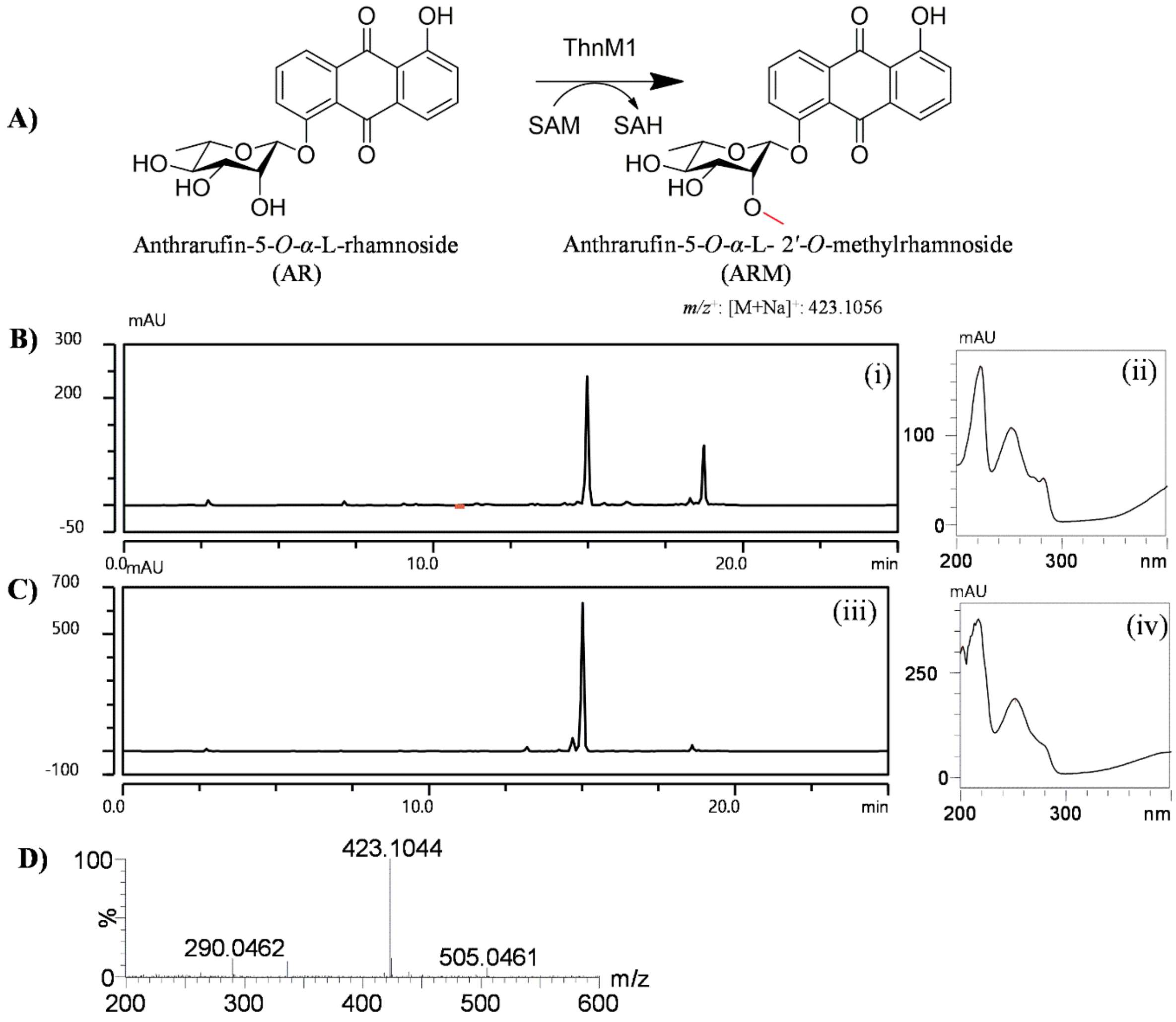

Figure S6.

A) Reaction scheme of methylation of anthrarufin-5-*O*-*α*-L-rhamnoside by ThnM1 in the presence of SAM at 40ºC for 3 h.

B) (i) HPLC-PDA chromatogram of reaction mixture of anthrarufin-5-*O*-*α*-L-rhamnoside with ThnM1 (ii) UV/VIS of methylated product of anthrarufin-5-*O*-*α*-L-rhamnoside.

C) (iii) HPLC-PDA chromatogram and (iv) UV/VIS of anthrarufin-5-*O*-*α*-L-rhamnoside Std.

D) HR-QTOF ESI/MS analysis of methylated product of anthrarufin-5-*O*-*α*-L-rhamnoside.

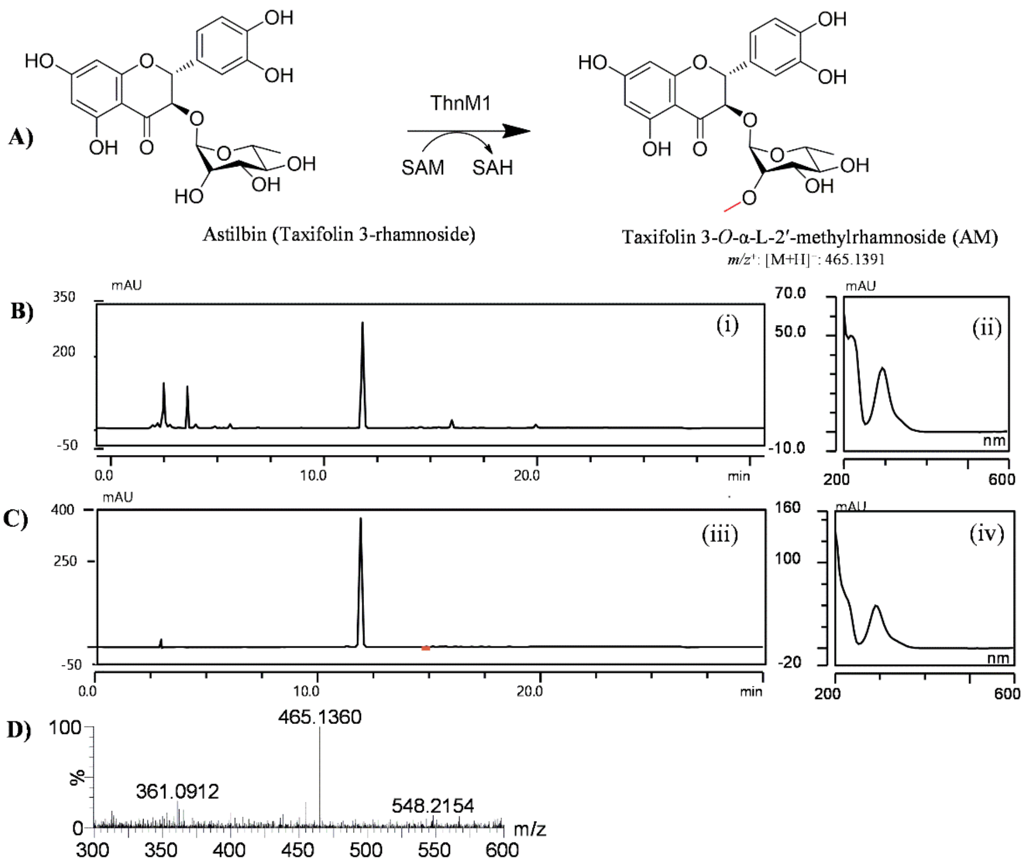

Figure S7.

A) Reaction scheme of methylation of astilbin by ThnM1 in the presence of SAM at 40ºC for 3 h.

B) (i) HPLC-PDA chromatogram of reaction mixture of astilbin with ThnM1 (ii) UV/VIS of methylated product of astilbin.

C) (iii) HPLC-PDA chromatogram and (iv) UV/VIS of astilbin Std.

D) HR-QTOF ESI/MS analysis of methylated product of astilbin.

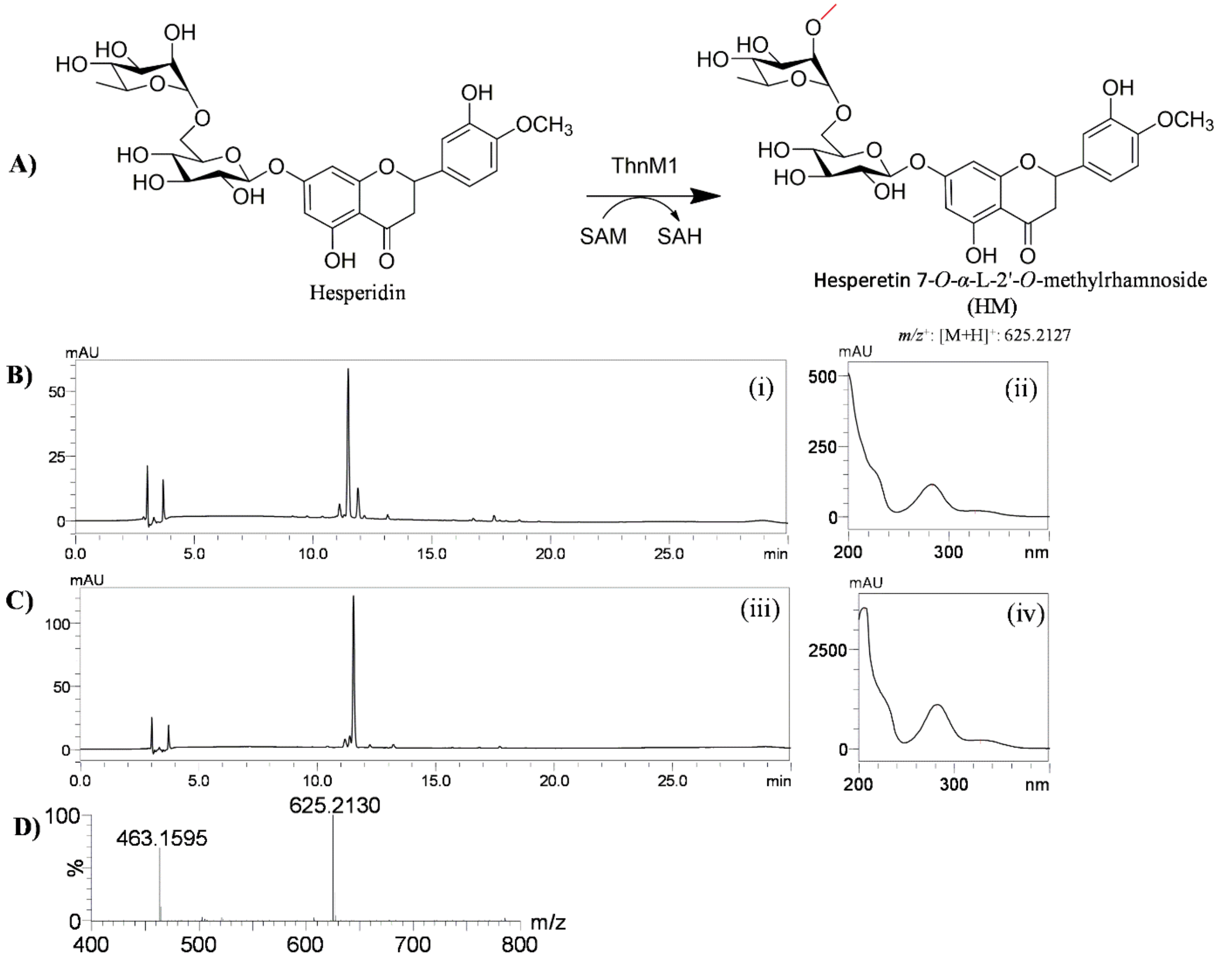

Figure S8.

A) Reaction scheme of methylation of hesperidin by ThnM1 in the presence of SAM at 40ºC for 3 h.

B) (i) HPLC-PDA chromatogram of reaction mixture of hesperidin with ThnM1 (ii) UV/VIS of methylated product of hesperidin

C) (iii) HPLC-PDA chromatogram and (iv) UV/VIS of hesperidin Std.

D) HR-QTOF ESI/MS analysis of methylated product of hesperidin

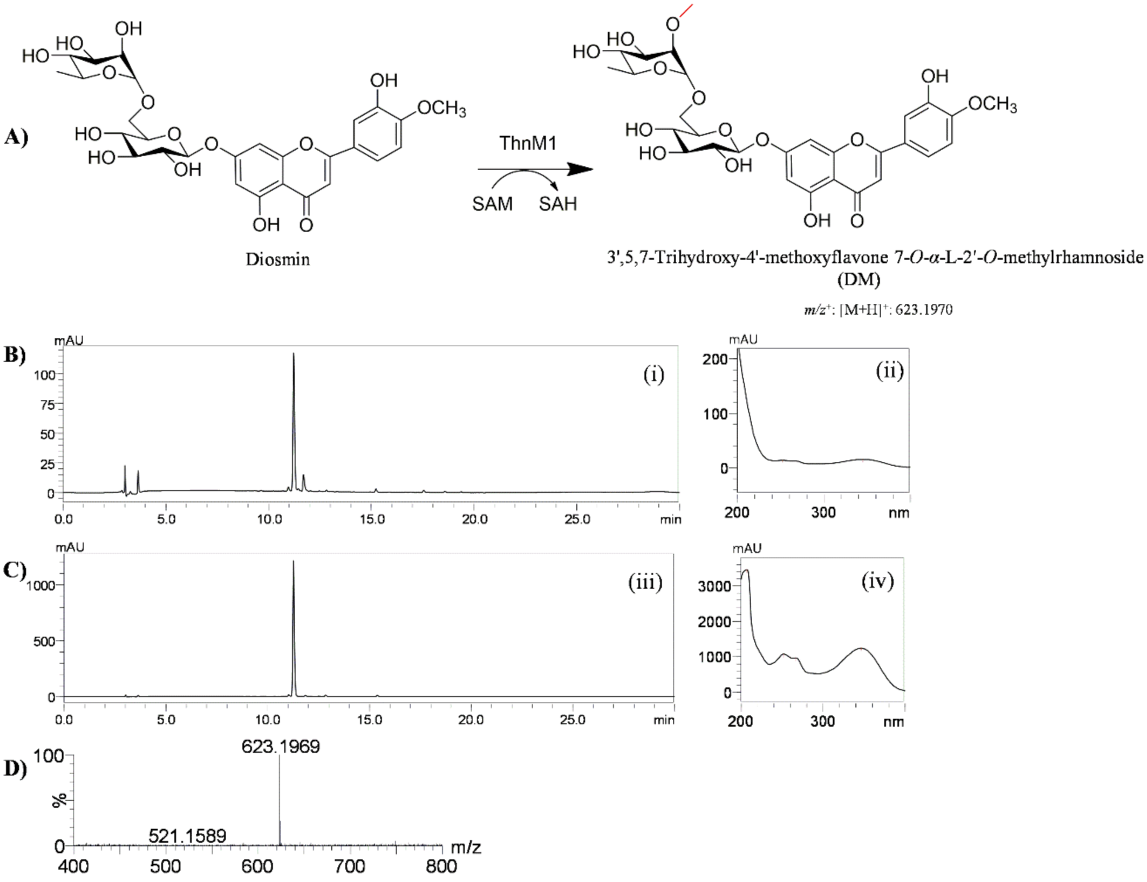

Figure S9.

A) Reaction scheme of methylation of diosmin by ThnM1 in the presence of SAM at 40ºC for 3 h.

B) (i) HPLC-PDA chromatogram of reaction mixture of diosmin with ThnM1 (ii) UV/VIS of methylated product of diosmin C) (iii) HPLC-PDA chromatogram and (iv) UV/VIS of diosmin Std.

D) HR-QTOF ESI/MS analysis of methylated product of diosmin.

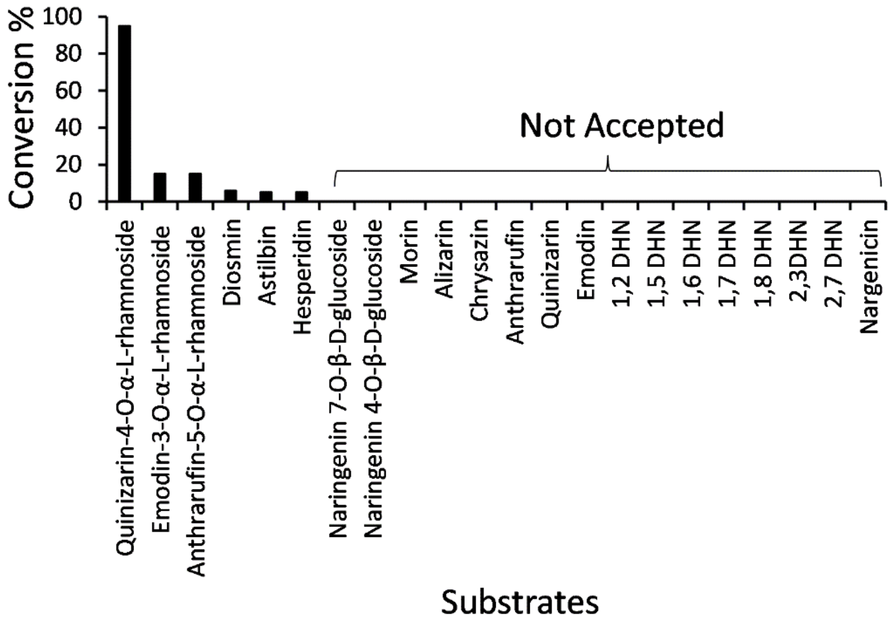

Figure S10.

Percentage conversion of all different substrates used in this study. The percent conversion was calculated by dividing the integrated peak area of a product by the sum of integrated peak area of product and substrate.

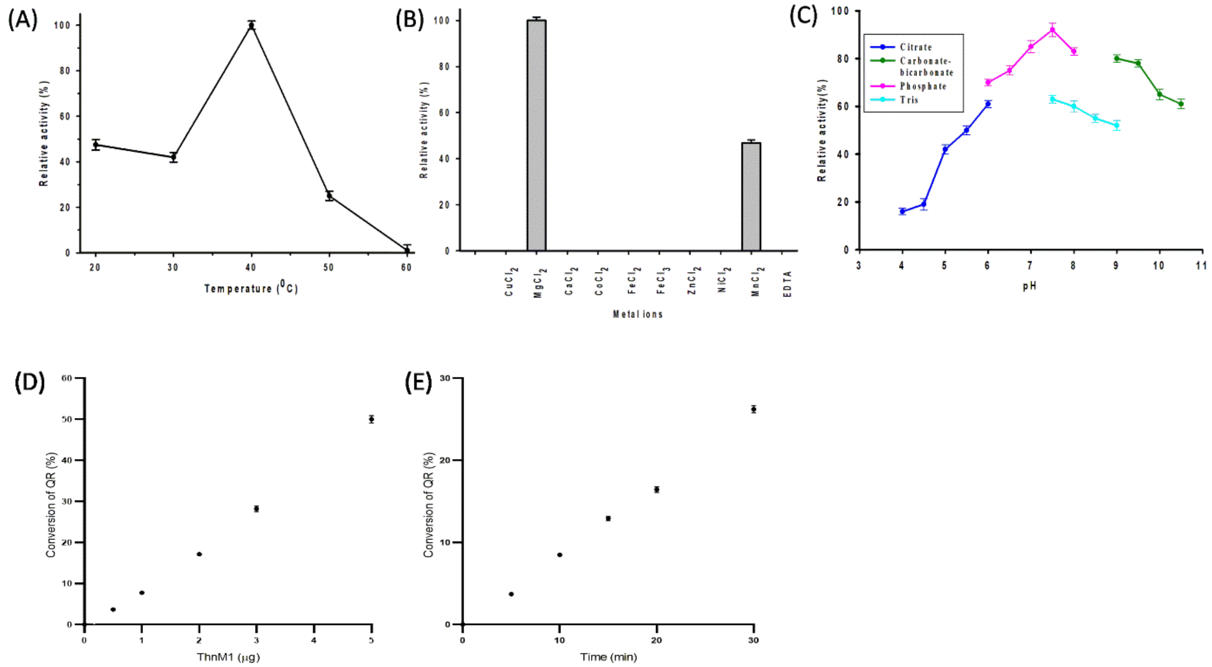

Figure. S11

Probing of ThnM1 assay conditions and determination of conditions for measuring initial velocity. A) Effect of different temperatures on the activity of purified ThnM1. B) Effect of pH on the activity of purified ThnM1. C) Different metal ions on the activity of purified ThnM1. D) ThnM1 assays with different enzyme concentrations. Assays comprising QR (5 µM), SAM (2 mM), $MgCl_2$ (2 mM) were performed in Tris-HCl buffer (50 mM, pH 7.5) with varying [ThnM1] (0.5-5 µg) at 40°C for 30 min. E) A time course of ThnM1 assay comprising QR (5 µM), ThnM1 (2 µg), SAM (2 mM), $MgCl_2$ (2 mM) were performed in Tris-HCl buffer (50 mM, pH 7.5) at 40°C within 30 min.

mycinamicin VI

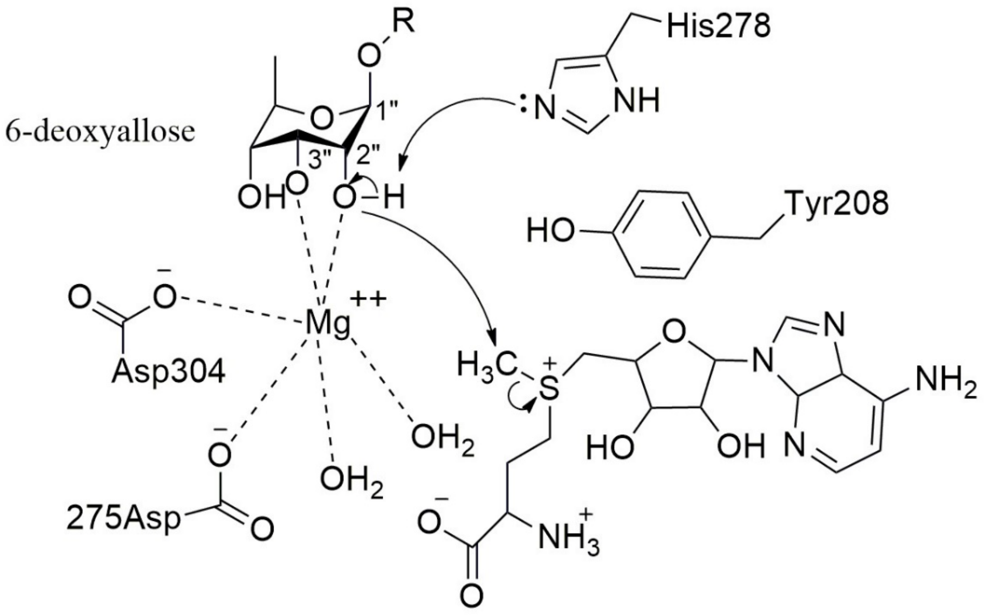

Figure S12.

Proposed reaction mechanism of MycE in natural product sugar methoxide biosynthesis [5].

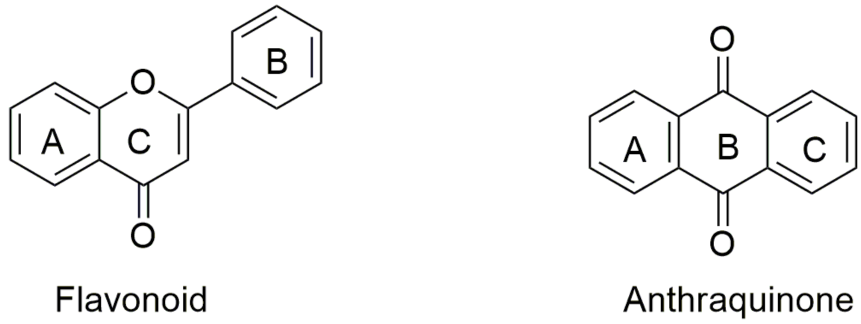

Figure S13.

Ring structure of flavonoid and anthraquinone.

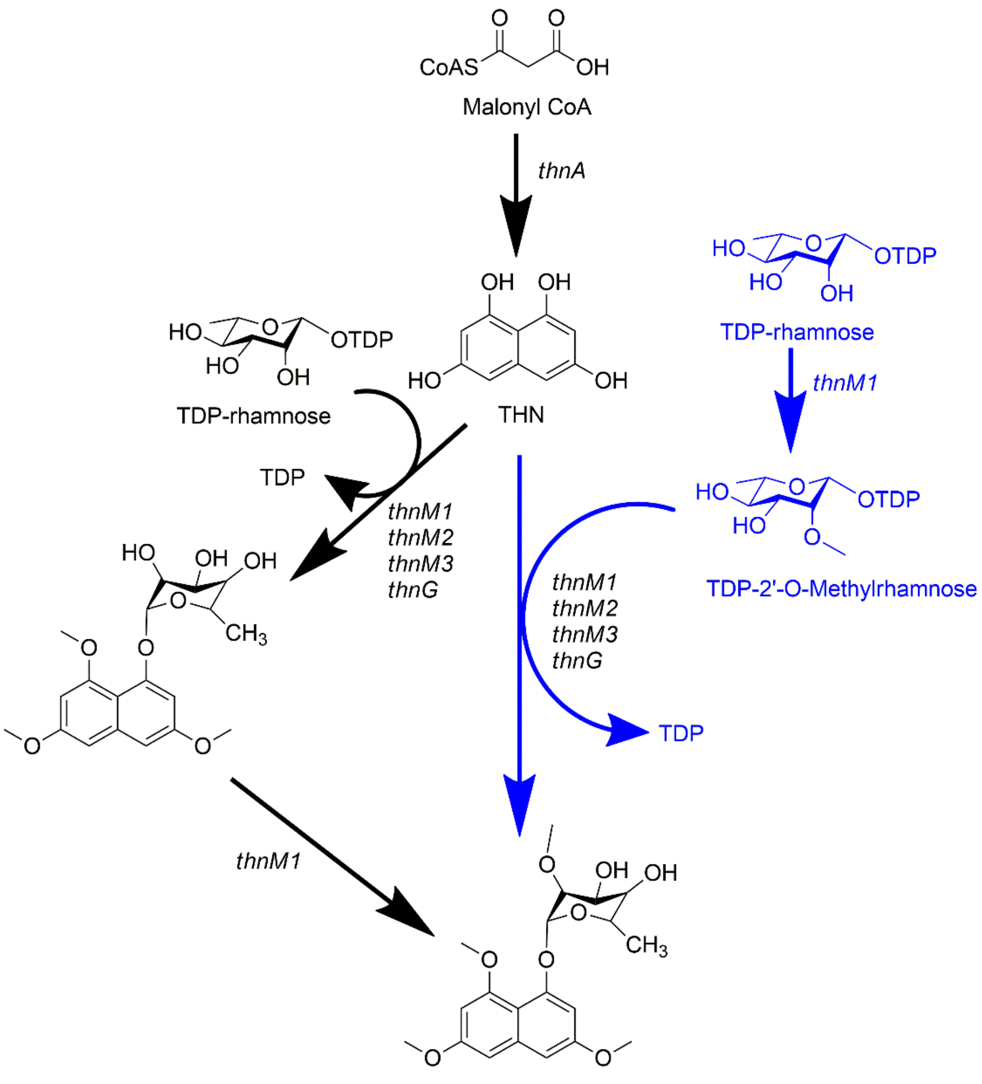

Figure S14.

Two possible approaches for the generation of 1-(α-L-(2-*O*-methyl)- 6-deoxymanno-pyranosyloxy)-3,6,8- trimethoxy naphthalene from THN using TDP-rhamnose and ThnM1. The pathway in blue arrow shows the generation of methylated NDP-sugar and eventual transfer of sugar moiety to aglycone by a glycosyltransferase enzyme. The pathway in black arrows shows the conjugation of a sugar molecule to a core metabolite followed by modification of sugar by a sugar methyltransferase.

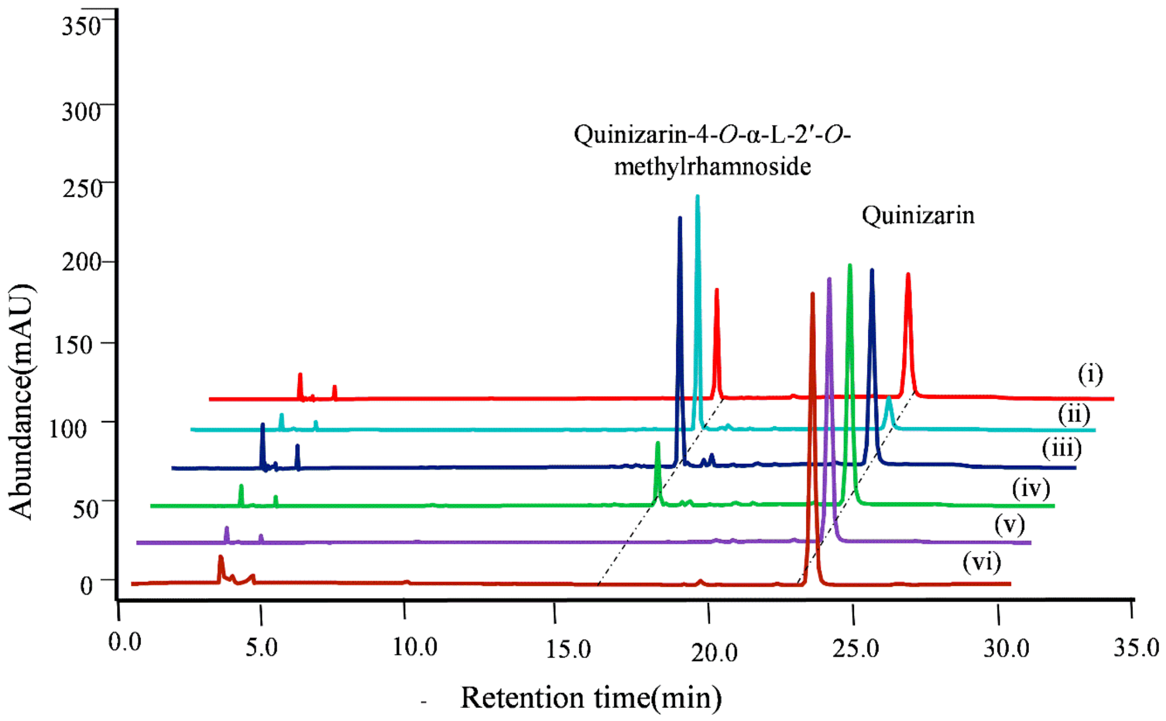

Figure S15.

HPLC-PDA analyses of whole cell bioconversion reaction mixture of quinizarin to quinizarin-4-*O*-*α*-L-methylrhamnoside by feeding different concentration of substrate. (i) 2mM quinizarin, (ii) 4mM quinizarin, (iii) 8mM quinizarin, (iv) 10mM quinizarin, (v) 12mM quinizarin, and (vi) 16mM quinizarin

NOTE: The numbering in the structure and spectrum is used for the annotation of the peaks only. The numbers in the below all NMR structure do not represent the exact position of carbon and functional groups

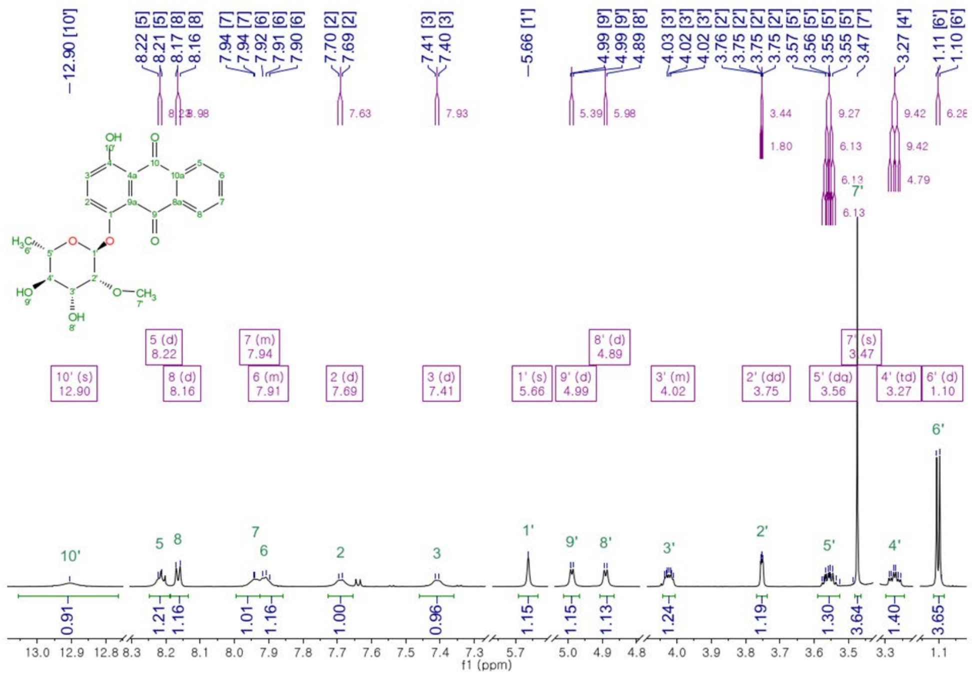

Figure S16.

(a) ^{1}H NMR spectrum of quinizarin-4-*O*-*α*-L-2′-*O*-methylrhamnoside at 700MHz in DMSO-d_6

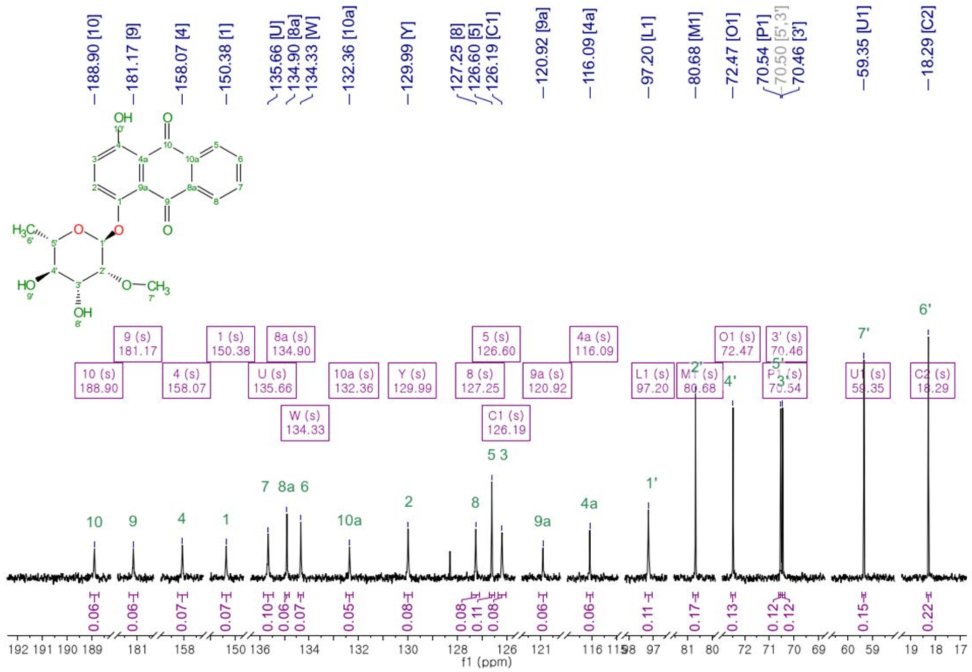

(b) ^{13}C NMR spectrum of quinizarin-4-*O*-*α*-L-2′-*O*-methylrhamnoside at 176 MHz in DMSO-d_6

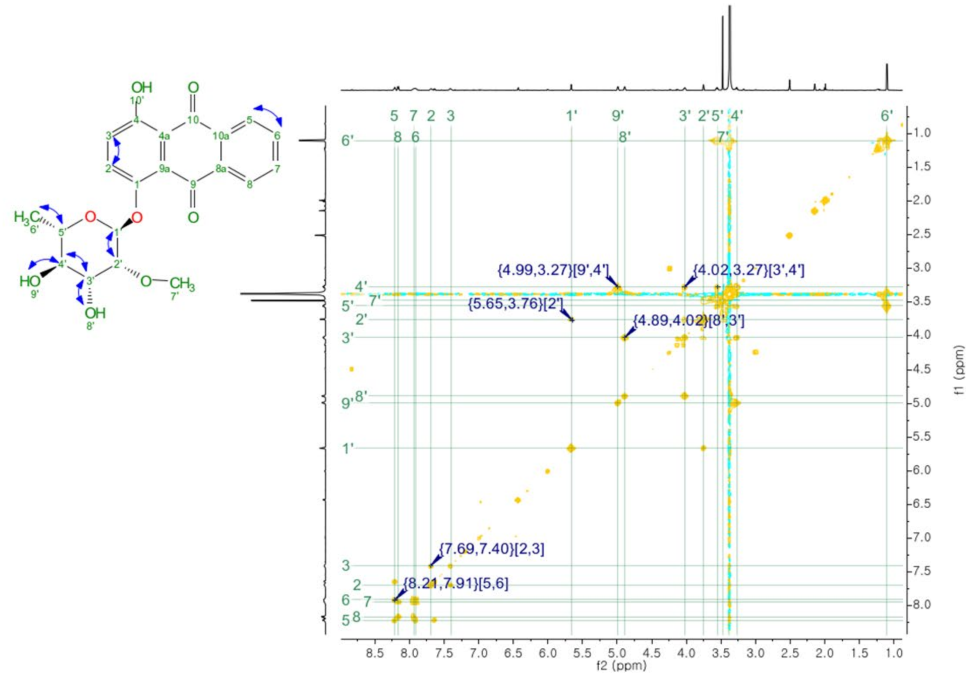

(c) ^{1}H-^{1}H COSY NMR of quinizarin-4-*O*-*α*-L-2′-*O*-methylrhamnoside

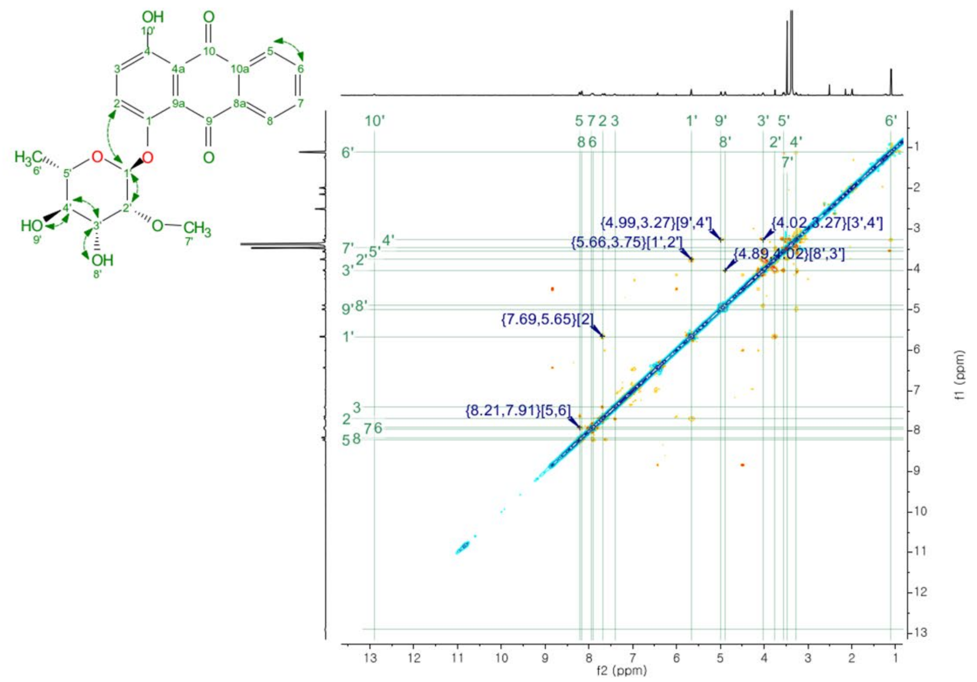

(d) ^{1}H-^{1}H ROSEY NMR of quinizarin-4-*O*-*α*-L-2′-*O*-methylrhamnoside

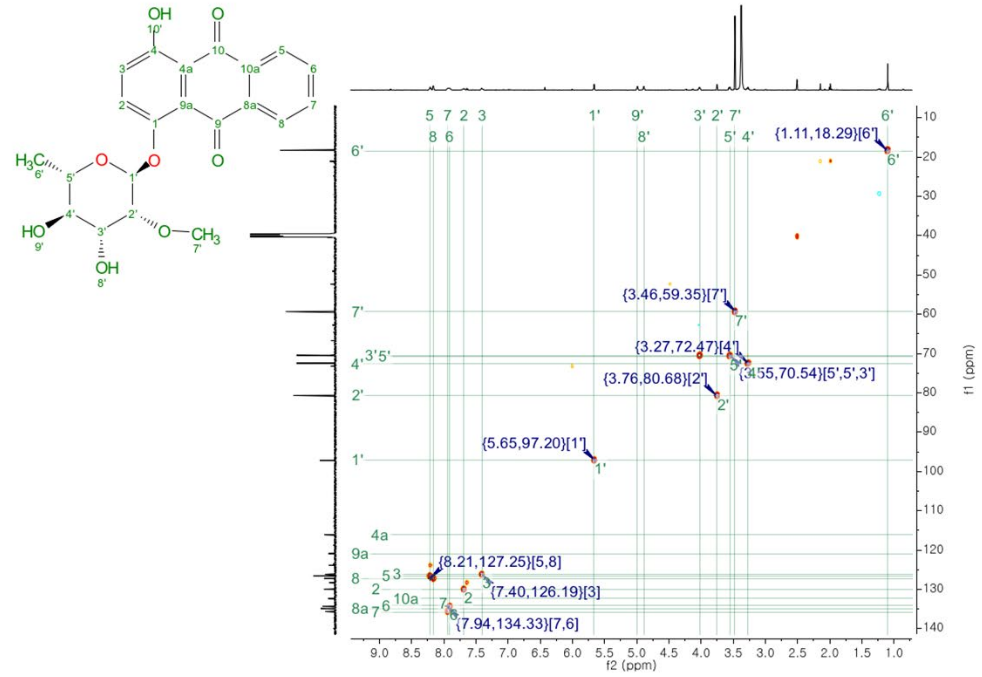

(e) ^{1}H-^{13}C HSQC-DEPT NMR of quinizarin-4-*O*-*α*-L-2′-*O*-methylrhamnoside

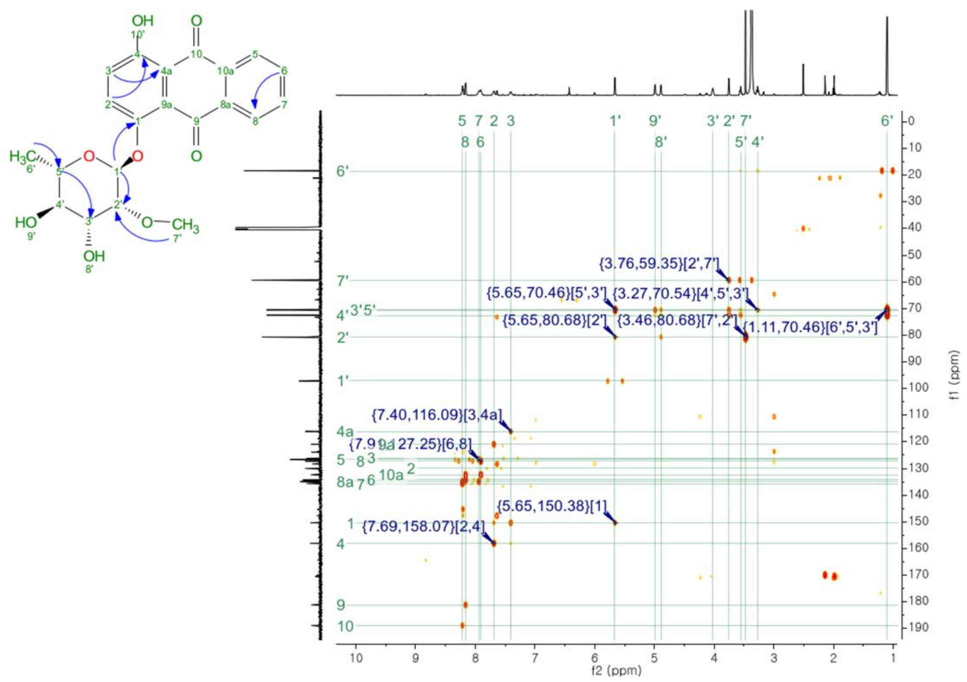

(f) ^{1}H-^{13}C HMBC NMR of quinizarin-4-*O*-*α*-L-2′-*O*-methylrhamnoside.

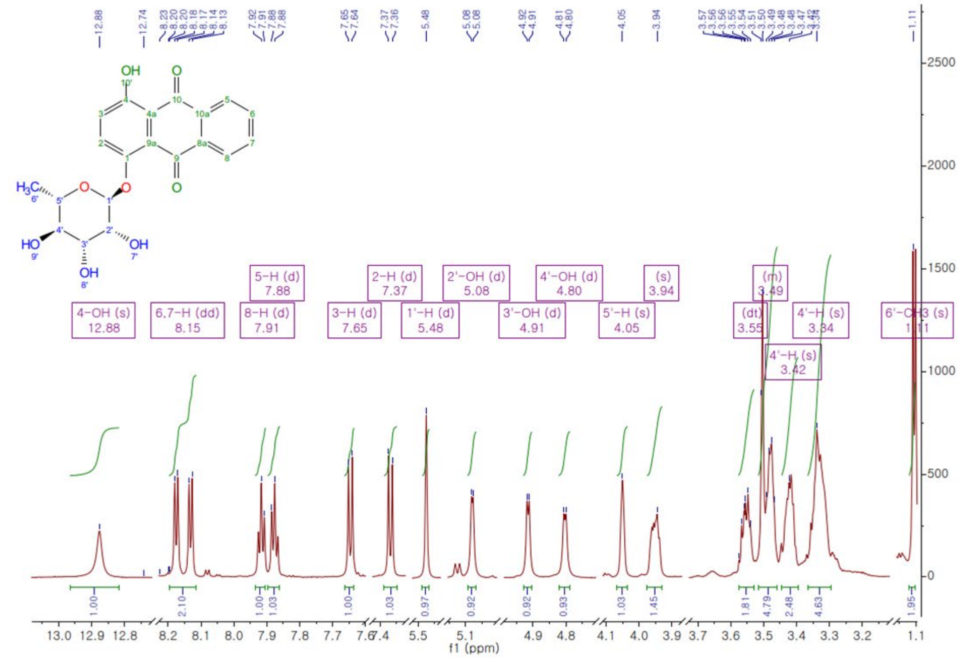

Figure S17.

(a) ^{1}H NMR spectrum of quinizarin-4-*O*-α-L-rhamnoside at 800MHz in DMSO-d$_6$

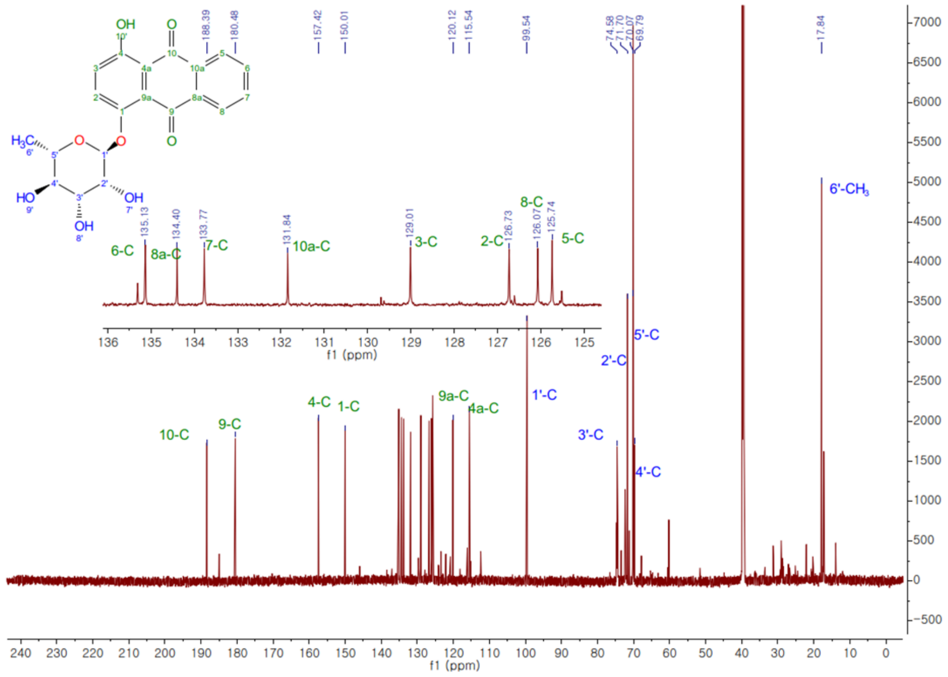

(b) ^{13}C NMR spectrum quinizarin-4-*O*-*α*-L-rhamnoside in 176MHz, DMSO-d6.

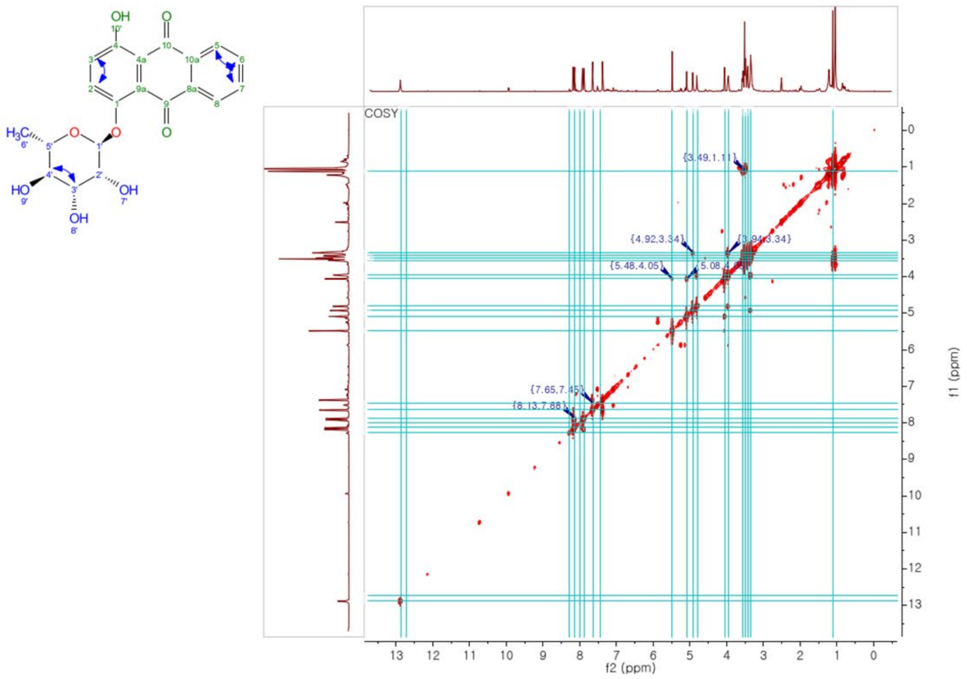

(c) ^{1}H-^{1}H COSY NMR of quinizarin-4-*O*-*α*-L-rhamnoside.

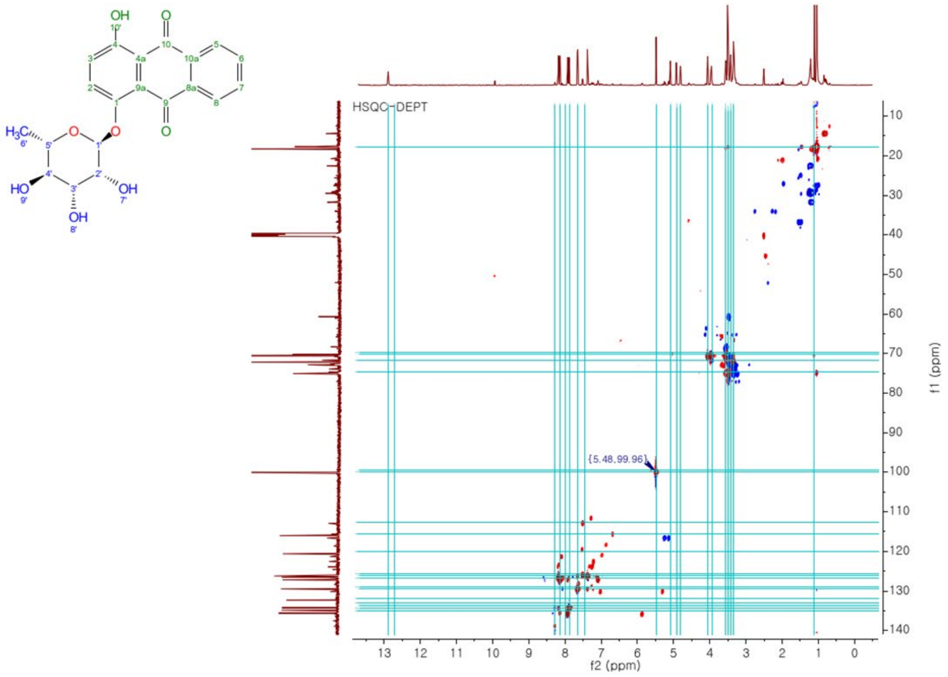

(d) ^{1}H-^{13}C HSQC-DEPT NMR of quinizarin-4-*O*-α-L-rhamnoside.

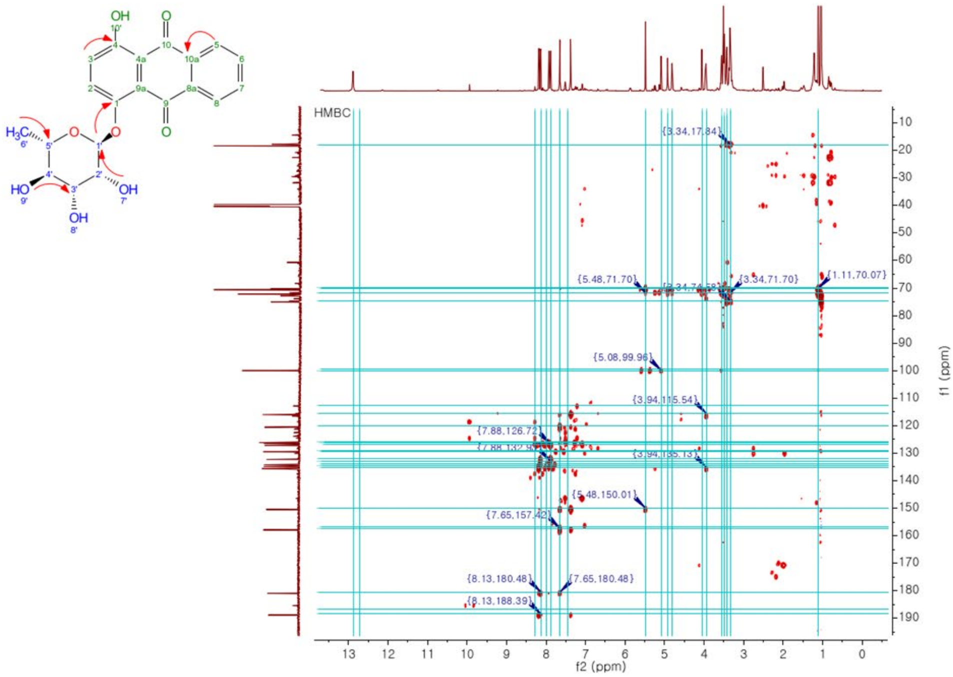

(e) ^{1}H-^{13}C HMBC NMR of quinizarin-4-*O*-*α*-L-rhamnoside.

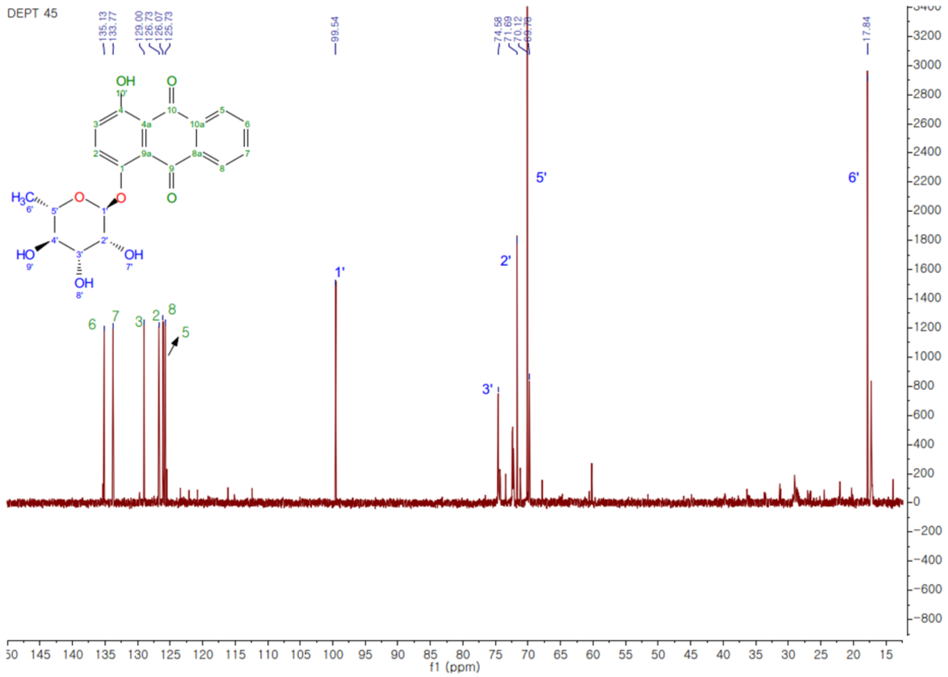

(f) DEPT 45 NMR spectrum of quinizarin-4-*O*-*α*-L-rhamnoside.

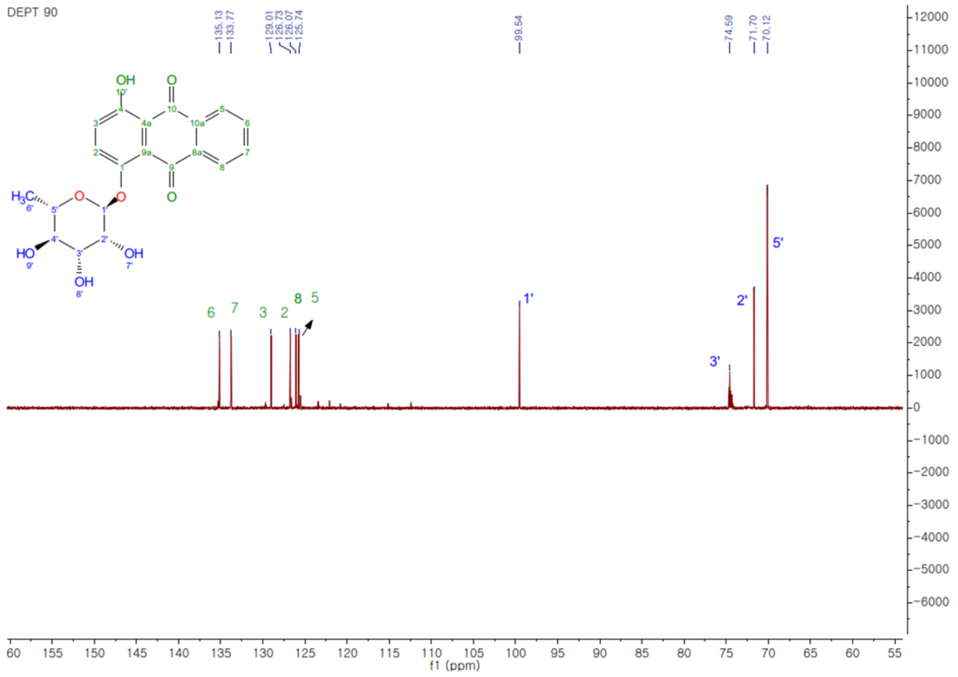

(g) DEPT 90 NMR spectrum of quinizarin-4-*O*-α-L-rhamnoside.

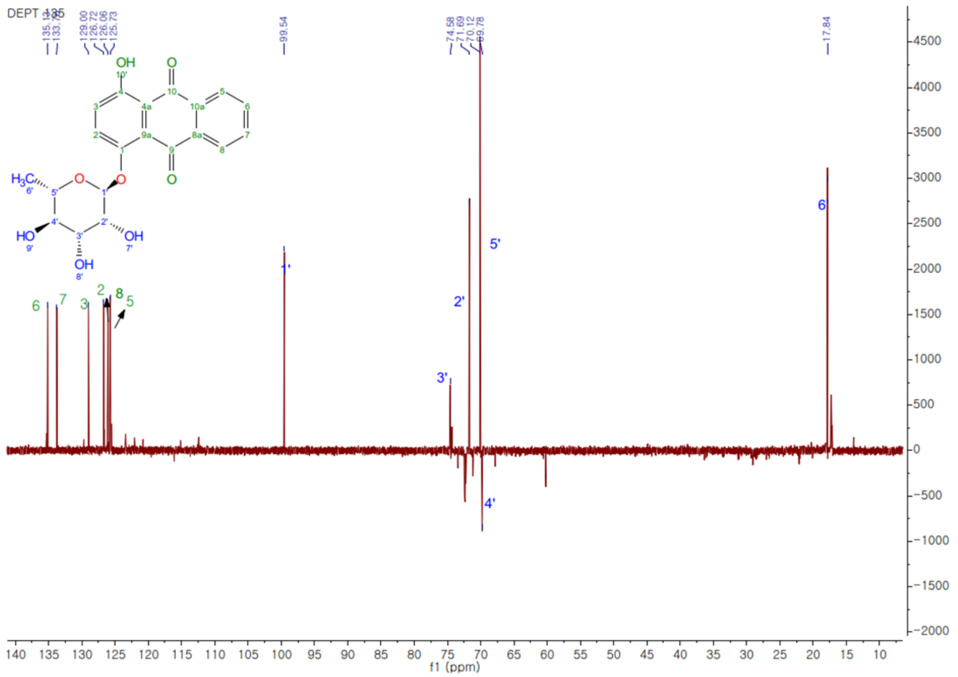

(h) DEPT 135 NMR spectrum of quinizarin-4-*O*-*α*-L-rhamnoside.

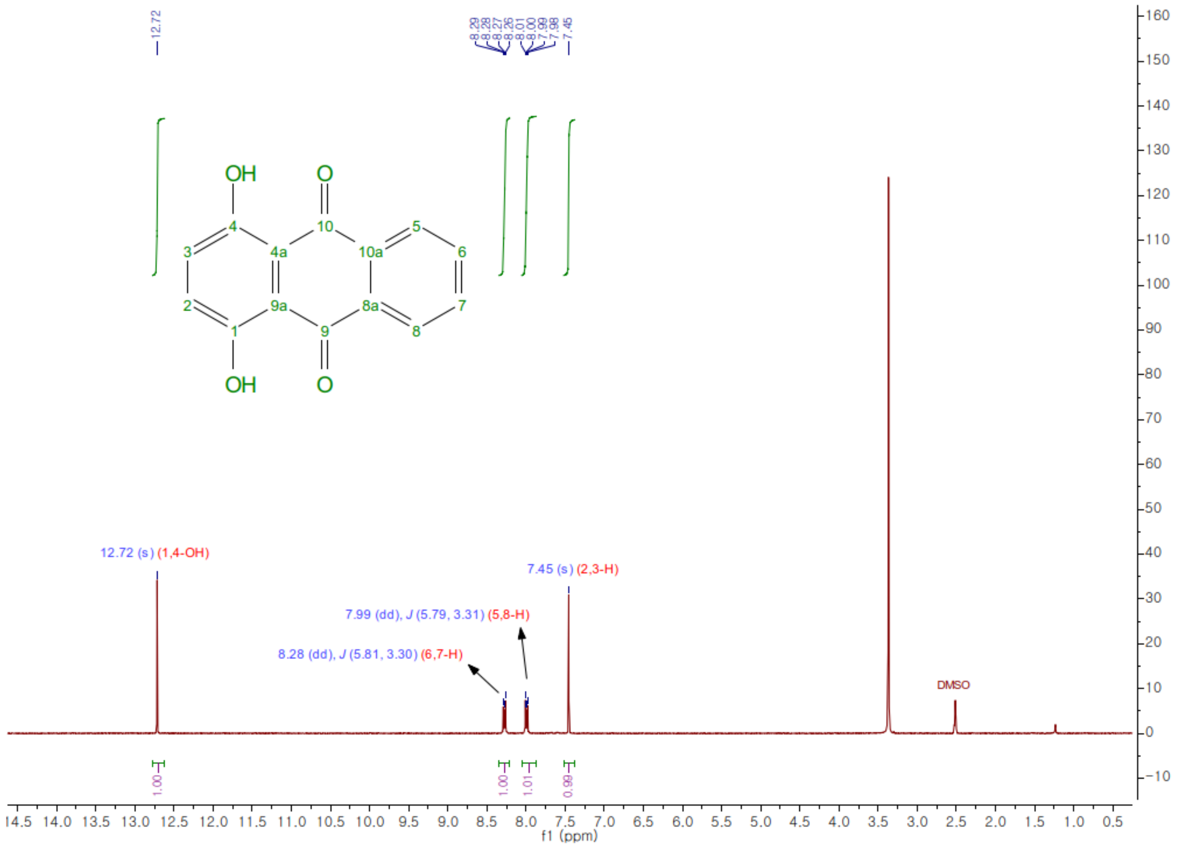

Figure S18.

(a) ^{1}H NMR spectrum of quinizarin at 300MHz in DMSO-d_6.

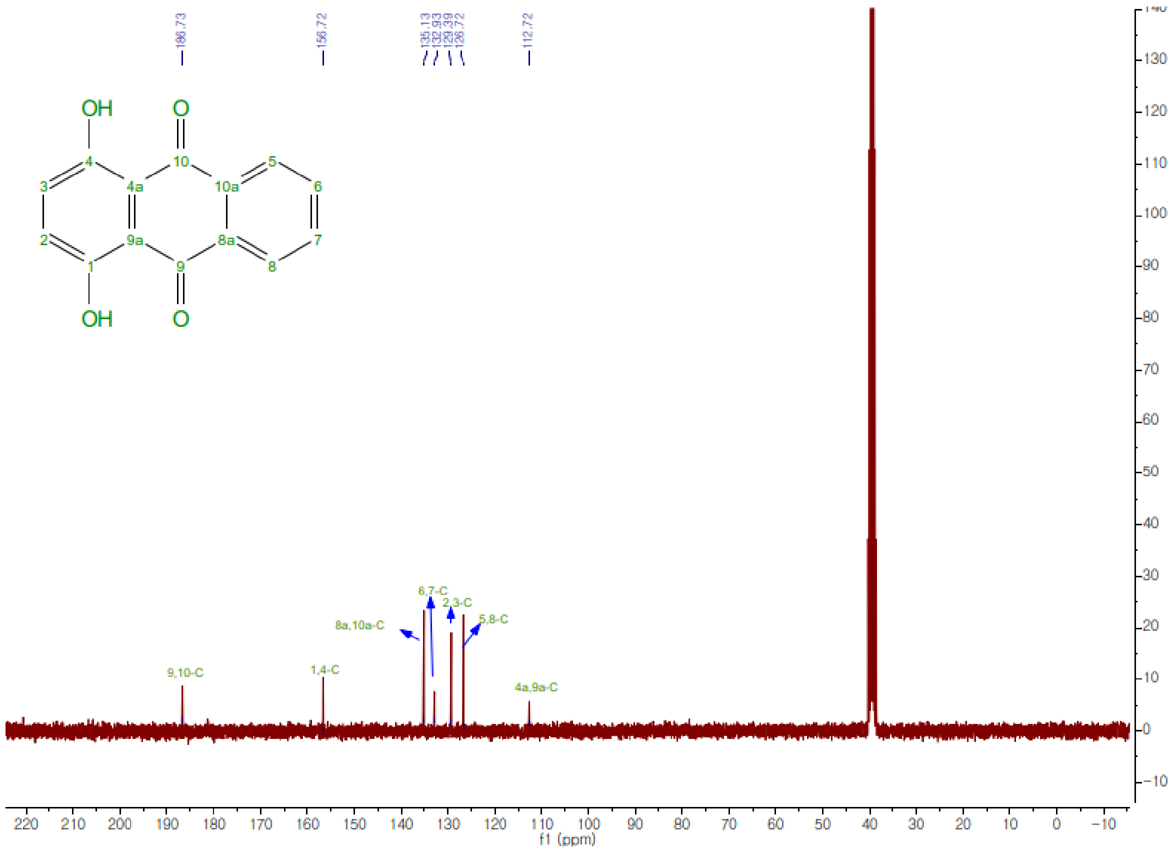

(b) ^{13}C NMR spectrum of quinizarin-4-*O*-*α*-L-rhamnoside at 176 MHz in DMSO-d_6.

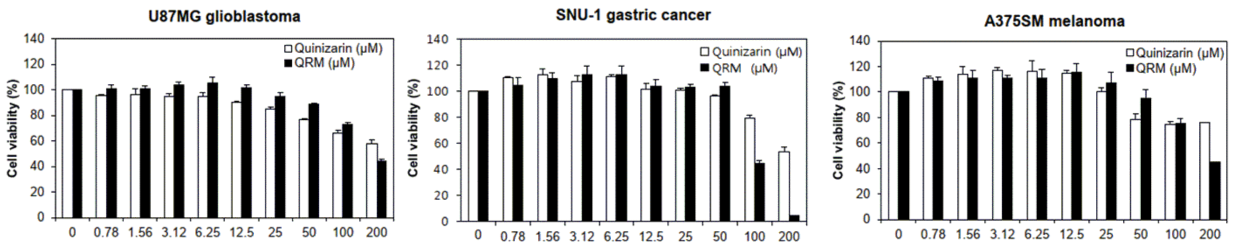

Figure S19.

Cell cytotoxicity assay of quinizarin and quinizarin-4-*O*-*α*-L-2′-*O*-methylrhamnoside. Cells were treated with various concentrations (0.0 ～ 200μM) of each compound.

References

1. Nguyen TTH, Shin HJ, Pandey RP, Jung HJ, Liou K, Sohng JK. 2020. Biosynthesis of Rhamnosylated Anthraquinones in *Escherichia coli.* J. Microbiol. Biotechnol. 30**:**398–403.

2. Parajuli P, Pandey RP, Trang NTH, Chaudhary AK, Sohng JK. 2015. Synthetic sugar cassettes for the efficient production of flavonol glycosides in *Escherichia coli*. Microb. Cell Fact. 14**:**1–12.

3. Jones DT, Taylor WR, Thornton JM. 1992. The rapid generation of mutation data matrices. Bioinformatics 8**:**275–282.

4. Kumar S, Stecher G, Li M, Knyaz C, Tamura K. 2018. MEGA X: Molecular evolutionary genetics analysis across computing platforms. Mol Biol Evol 35**:**1547–1549.

5. Akey DL, Li S, Konwerski JR, Confer LA, Bernard SM, Anzai Y, Kato F, Sherman DH, Smith JL. 2011. A new structural form in the SAM/metal-dependent O-methyltransferase family: MycE from the mycinamicin biosynthetic pathway. J Mol Biol 413**:**438–450.
